# An agentic AI framework connecting language models to electronic health records and a biomedical knowledge graph for real-world evidence

**DOI:** 10.3389/frai.2026.1883853

**Published:** 2026-09-04

**Authors:** Gianmarco Bellucci, Wanjun Gu, Peter W. Rose, Sergio E. Baranzini

**Affiliations:** 1 Department of Neurology, Weill Institute for Neurosciences, University of California San Francisco, San Francisco, CA, United States; 2 Graduate Program in Biomedical Informatics, University of California San Francisco, San Francisco, CA, United States; 3 San Diego Supercomputer Center, University of California San Diego, La Jolla, CA, United States

**Keywords:** agentic artificial intelligence, biomedical knowledge graph, electronic health records, large language models, Model Context Protocol, real-world evidence

## Abstract

**Background:**

Accessing large-scale clinical and biomedical databases remains a significant barrier for clinicians and researchers, requiring substantial computational expertise. Agentic artificial intelligence frameworks, in which large language models (LLMs) orchestrate multi-step reasoning and query execution under interactive human supervision, offer the potential to democratize data access and accelerate evidence generation.

**Methods:**

We applied the Model Context Protocol (MCP) to integrate, within a single agentic workflow, an Observational Medical Outcomes Partnership (OMOP)-standardized electronic health record (EHR) database from an academic health system (>7 million subjects), with the Scalable Precision Medicine Open Knowledge Engine (SPOKE), a curated knowledge graph integrating relationships biomedical concepts from expert-maintained resources. Using this implementation (MedCP), we evaluated the approach across 100 benchmarking clinical research tasks, 617 biomedical factual accuracy questions (BiomixQA), an integrative case study linking clinical co-occurrence with molecular similarity, and a standardized protocol for generating and replicating real-world studies.

**Results:**

Across the 100-task benchmark, knowledge-graph access improved mean scores overall for GPT-5.5 and Claude Opus 4.8, though the pattern differed between models; on BiomixQA, SPOKE grounding raised multiple-choice accuracy for both models, without changing true/false accuracy. In the case study, disease co-occurrence patterns extracted from the EHR correlated with molecular network similarity, surfacing mechanistic hypotheses from real-world data. Applied to the replication of published observational studies, the research protocol compressed timelines from months to hours, lowering the technical barrier to query generation and execution while study design and interpretation remained under expert supervision.

**Conclusions:**

An agentic AI infrastructure that combines institutional EHR data with curated biomedical knowledge via MCP can serve as a transparent, domain-grounded, supervised research assistant for real-world evidence, supporting both hypothesis generation and testing.

## Introduction

Healthcare is among the most data-rich and complex fields in our society, yet much of this information remains underutilized by clinicians and researchers who could translate it into better diagnoses, treatment regimens, and cost savings (Davenport and Kalakota, 2019). Electronic health records (EHRs) and curated biomedical databases capture millions of structured data points but accessing them typically requires fluency in domain-specific query languages, creating a barrier that slows discovery and limits the real-time use of real-world data. Even when data are available, the gap between clinical questions and computational implementation is wide enough that only an estimated 15% of clinical trials published in high-impact journals could feasibly be replicated with currently accessible real-world data (Bartlett et al., 2019), underscoring the persistent friction between clinical research questions and the resources needed to answer them.

Large language models (LLMs) have shown remarkable ability to understand and generate medical text, fueling enthusiasm for their use in clinical practice and translational research (Singhal et al., 2023; Tu et al., 2024). Recent advances extend these capabilities toward agentic systems. By *agentic AI* we mean a LLM that interleaves reasoning with external actions: planning a task, querying external sources, calling and using proper tools, and iteratively refining its answer within a single workflow, rather than returning a single response to a prompt. This paradigm was first formalized as ReAct (Yao et al., 2023). Such agents additionally maintain contextual memory across interactions and can orchestrate complex workflows in a goal-directed fashion (Xu and Sankar, 2025). Empirical taxonomies of healthcare agent implementations suggest that external knowledge integration is now commonly realized, while safety-critical capabilities such as drift detection and treatment planning remain largely unaddressed (Vatsal et al., 2026), and dedicated benchmarks built on virtual EHR environments confirm that even the strongest current models leave substantial room for improvement on realistic clinical agent tasks (Jiang et al., 2025). However, their direct application in high-stakes environments is still limited by concerns about accuracy, reproducibility, and transparency (Kim et al., 2025; Mesinovic et al., 2025).

These challenges highlight the importance of grounding AI models in structured, trustworthy biomedical sources. Recent studies emphasize that integrating external knowledge can bolster an LLM's clinical reasoning and reduce errors. For example, enriching LLMs with a knowledge graph (KG) improved factual accuracy and reasoning consistency in benchmark studies (Gao Y. et al., 2025), and KG-enhanced frameworks have shown particularly large gains on complex biomedical question-answering tasks where LLM parametric knowledge is unreliable (Feng et al., 2025). Biomedical knowledge graphs like Scalable Precision Medicine Open Knowledge Engine (SPOKE) encode scientifically vetted relationships that an LLM can draw on, rather than relying solely on parametric memory. Similarly, EHRs contain extensive structured and semi-structured patient data that, if safely and flexibly integrated, could strengthen population-level analyses and accelerate hypothesis testing. Combining EHR-derived context with curated molecular and pharmacological knowledge allows LLMs to move beyond generic responses toward data-driven insights relevant to study design and translational research.

Efforts to operationalize this integration have often relied on retrieval-augmented generation (RAG), in which models fetch external evidence at inference time (Matsumoto et al., 2024; Soman et al., 2024); iterative variants that allow LLMs to refine retrieval through follow-up queries have further improved performance on complex medical questions (Xiong et al., 2025). However, many existing solutions rely on bespoke integrations or *ad-hoc* pipelines that are not standardized, making them difficult to scale across healthcare systems. Moreover, most RAG implementations depend on large vector databases, which require careful tuning, storage optimization, and domain-specific embedding strategies to ensure relevance and performance–introducing additional operational complexity and limiting generalizability.

The Model Context Protocol (MCP) was introduced as an open standard for connecting AI models to external tools and data through a uniform interface. The protocol is often compared to a USB-C port for AI applications: just as USB-C provides a standardized way to connect devices to peripherals, MCP provides a standardized way to connect an AI application to external tools and data (Model Context Protocol, 2025). Concretely, MCP specifies the transport, the way a client and server establish which features each supports, and the schema by which a server advertises the tools and resources it exposes, so that adding a data source requires only implementing a conformant server, without modifying the model, the client, or any existing retrieval pipeline.

A recent perspective sets out the conceptual case for this architecture in clinical medicine, identifying interoperability, auditability, and security as the properties that will determine whether such systems are usable in practice, and noting that the argument remains largely conceptual, with production deployments scarce (Choi and Yoo, 2026).

Empirical work has begun to supply the missing evidence: MCP-based pipelines have been used to query Fast Healthcare Interoperability Resources (FHIR) resources for clinical decision support (Ehtesham et al., 2025), and a recent real-world evaluation showed that an LLM connected to a hospital EHR via MCP could complete most clinical information-retrieval tasks correctly (Masayoshi et al., 2025). These efforts point to MCP as a promising substrate for connecting LLMs to clinical data, but, to our knowledge, none has so far combined institutional EHR access with a curated biomedical knowledge graph in a single agentic workflow.

We report here the implementation and evaluation of such a workflow: MedCP is an MCP server that exposes to a single agent both the OMOP-formatted EHR of a large academic medical center and SPOKE, a curated biomedical knowledge graph integrating relationships among diseases, symptoms, genes, drugs, pathways and other biomedical entities assembled from dozens of expert-maintained sources (Morris et al., 2023).

The objective is to lower the technical barrier to real-world evidence generation, allowing a clinician or researcher to interrogate institutional clinical data through conversational interaction while grounding the resulting analyses in expert-curated biomedical knowledge rather than in the parametric memory of the model.

To assess whether this approach generalizes across the spectrum of different clinical and translational research tasks, we evaluated it on a set of benchmarking questions spanning major medical domains and increasing in complexity. We then applied the same pipeline to two real-world evidence tasks: an integrative case study linking clinical co-occurrence with molecular similarity, and the generation and replication of real-world evidence studies. The system is designed to operate under expert supervision: it exposes the queries that produced each result, but the interpretation of those results remains the responsibility of the investigator. Our findings suggest that that MCP-enabled agentic AI can broaden access to real-world clinical data while preserving the auditability that clinical research requires, opening a practical route toward multi-institutional deployment on standardized data models.

## Materials and methods

### System architecture

**Figure 1:**
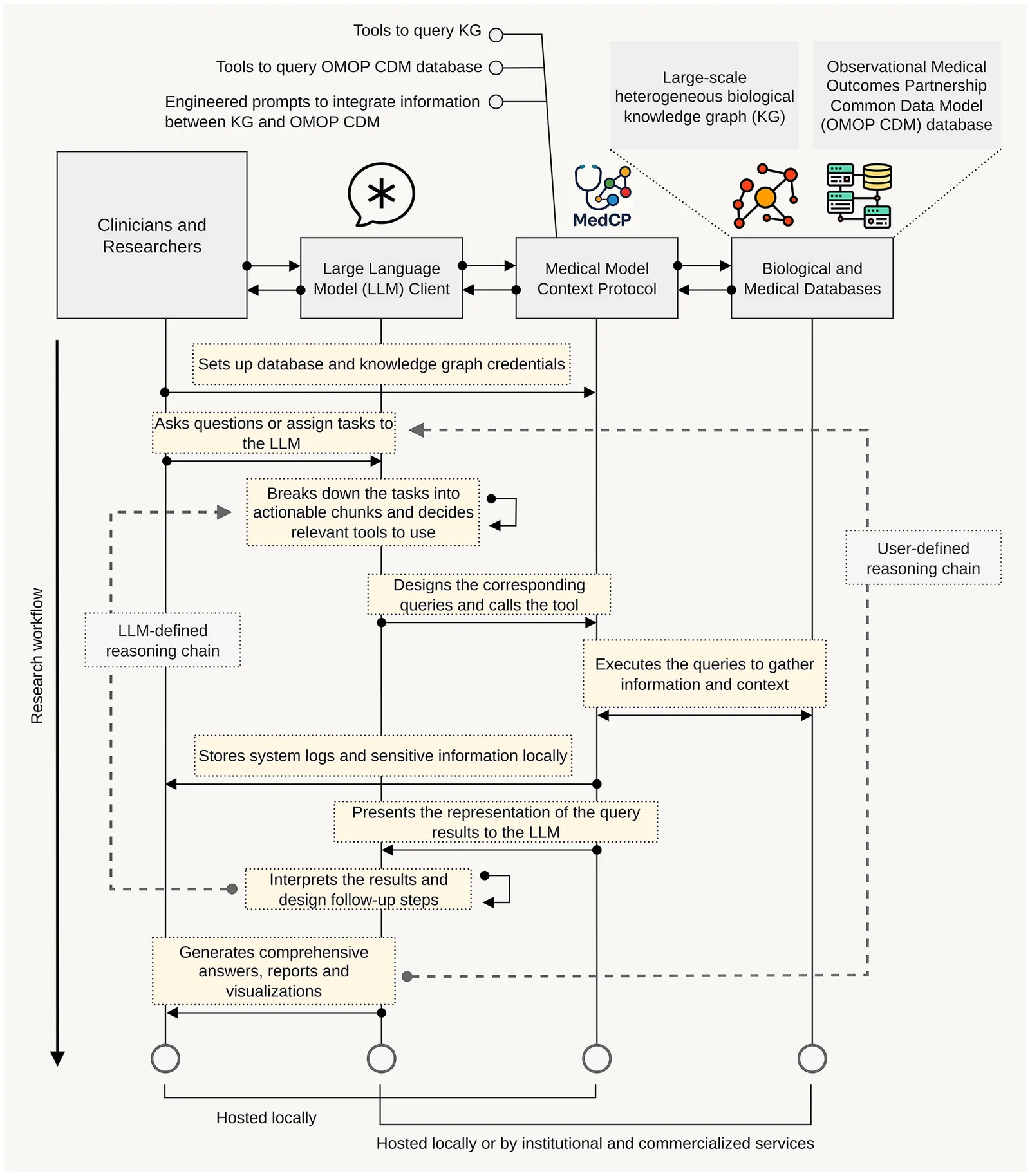
MedCP architecture and question-to-answer workflow. Clinicians and researchers interact with a large language model (LLM) “interpreter–planner” connected to MedCP, a model context protocol that exposes audited, read-only tools for institutional data access. After one-time local configuration of endpoints and credentials, the LLM translates user questions into schema-aware, parameterized SQL queries for the UCSF de-identified OMOP CDM EHR and Cipher queries for the SPOKE biomedical knowledge graph. MedCP executes these queries locally within the health system network, stores execution logs on the user's machine, and returns structured, minimal outputs (e.g., cohort counts/timelines from OMOP and typed concept-to-concept paths with provenance from SPOKE). The LLM iteratively interprets results, plans follow-up queries, and composes final responses as prose, tables, or visualizations. Dashed lines depict the reasoning loops (LLM-defined and user-directed), while the privacy boundary is maintained throughout: the LLM never holds database credentials, and only de-identified or aggregate results leave the underlying systems. Hosting of the LLM and MedCP may be local or via institutional/commercial services, with all database execution remaining local.

We evaluated the utility of the Model Context Protocol to aid the generation of real-world evidence in biomedicine by building MedCP, an agentic AI framework that connects an LLM to an EHR database and to a curated biomedical knowledge graph through audited, read-only tools that run on the user's machine inside the health system network (Figure 1). The framework has four sections:

1. First, clinicians and researchers authenticate with their institutional credentials (under a virtual private network connection, when required); all queries execute locally under the least-privileged roles.
2. Second, an LLM “interpreter–planner” translates questions into database queries.
3. Third, the MedCP toolkit (Table 1) exposes schema-aware tools and prompt templates that convert natural language into parameterized Structured Query Language (SQL) (for the clinical records) and Cipher (for the knowledge graph), returning only the minimum necessary results.
4. Fourth, MedCP connects to two resources: (i) a de-identified University of California, San Francisco (UCSF) electronic health record (EHR) database standardized to the Observational Medical Outcomes Partnership (OMOP) Common Data Model, and (ii) the Scalable Precision Medicine Open Knowledge Engine (SPOKE), a Neo4j-backed biomedical knowledge graph.

**Table 1:** Summary table of the tools and functions that MedCP uses for querying KG and EHR.

Tool	Backend	Purpose	Inputs	Output format
get_knowledge_graph_schema()	Neo4j (KG) via neo4j Python driver; requires APOC	Expose a compact, cleaned schema summary (nodes, properties, relationship types/directions) to guide Cipher design	No arguments	The function returns a JSON string representing a simplified schema object generated from the APOC schema call, including node and relationship types, optional counts, labels, indexed property types, and relationship directions.
query_knowledge_graph()	Neo4j (KG) via neo4j Python driver	Execute read-only Cipher for retrieval/inference across biomedical entities (e.g., drug–disease, protein interactions, pathways)	The Cipher query must not include any write operations such as MERGE, CREATE, SET, or DELETE. An optional parameters dictionary can be provided, which defaults to an empty dictionary if not specified.	The function returns a JSON string containing an array of record dictionaries, where each record lists field names and their corresponding values.
list_clinical_tables()	SQL Server (EHR) via pymssql	Enumerate BASE TABLES for schema discovery and query scaffolding	No arguments	The function returns a JSON string containing a list of objects with the schema name, table name, type, and full table name, obtained from the database's information schema and ordered by schema and table.
query_clinical_records()	SQL Server (EHR) via pymssql	Execute read-only SQL for clinical data retrieval	The SQL query must start with SELECT, WITH, or DECLARE, and cannot contain any write, data definition, or execution commands such as INSERT, UPDATE, DELETE, DROP, ALTER, or EXEC. Basic safeguards are applied to prevent injection patterns, including those that contain a semicolon followed by another command.	If the query produces results, the output is a CSV file with a header line followed by data rows. If there are no columns or rows returned, the function outputs a message indicating that the clinical query executed successfully but produced no results.

This table summarizes the core tools implemented in the integrated knowledge graph (KG) and electronic health record (EHR) query interface, detailing their computational backends, functional purposes, input requirements, and output formats. All tools were developed using Python 3.12, with neo4j-driver 5.21 for graph database connectivity and pymssql 2.3.0 for SQL Server access. The Neo4j components require the Awesome Procedures on Cipher (APOC) 5.11 library to extract schema metadata and perform optimized graph queries.

MedCP uses the official Python MCP server Application Programming Interface (API) in a dual-mode design that supports both traditional initialized sessions and sessionless request flows. This preserves compatibility with established clients while enabling stateless deployments and optional transport-level routing and authorization controls; the evaluated clinical-data entry point remained stdio-only.

Read-only access is enforced at three layers, providing defense-in-depth against both inadvertent and adversarial write attempts. At the prompt layer, tool schemas and system instructions direct the LLM to generate only read queries. At the connector layer, the MedCP server validates every query before it reaches the database: Cipher queries are scanned for write operations (MERGE, CREATE, SET, DELETE) and rejected if any are detected, while SQL queries are checked to ensure they begin with SELECT, WITH, or DECLARE, and are blocked if they contain INSERT, UPDATE, DELETE, DROP, ALTER, or EXEC statements. At the database account layer, the service accounts used by the pymssql and neo4j Python drivers are registered at the institutional database with read-only roles (SQL Server db_datareader for OMOP, Neo4j reader for SPOKE); any write attempt that bypassed the upper two layers would still fail at the database side, independently of the prompt or the connector validator.

Configuration (endpoints, credentials) and execution logs are stored locally to support reproducibility and audit while avoiding any transfer of protected health information outside institutional boundaries.

### Privacy and data governance

The UCSF OMOP instance queried in this application is pre-de-identified according to the Health Insurance Portability and Accountability Act (HIPAA) Safe Harbor method before being made available to authorized researchers; the framework itself does not perform de-identification at run-time but operates exclusively on this already de-identified layer. Large language models are accessed through the UCSF Versa API Gateway, an institutional platform covered by a Business Associate Agreement (BAA) between UCSF and providers: queries are routed through a UCSF on-premises Amazon Web Services (AWS) Bedrock endpoint so that prompt and tool-call traffic does not leave the institutional network. Only de-identified or aggregate query results are returned to the LLM, and no patient identifiers, free-text notes, or raw record content are exchanged with external services.

### Models and inference

The framework is model-agnostic in its architecture: MedCP exposes tools through the Model Context Protocol and any MCP-capable client can invoke them. The empirical results reported here reflect the performances of the LLM used. Specifically, we evaluated GPT-5.5 and Claude Opus 4.8 (OpenAI GPT-5.5; Anthropic Claude Opus 4.8) on the 100 benchmarking questions, and on BiomixQA questions (see below) under both MedCP configurations. The case study analyses employed Claude Opus 4.5. The scores, token counts and failure modes reported below are properties of these models in this deployment. The LLMs used in this study were applied in their original, pre-trained form, without fine-tuning, hyperparameter adjustment, or task-specific training. MedCP relies on these general-purpose LLMs to coordinate multi-step reasoning and tool use; all model behavior is controlled through system prompts and tool definitions provided by the MCP framework, with no modification to model weights or inference settings.

### Clinical database

We evaluated MedCP against the de-identified UCSF OMOP instance, which contains longitudinal EHR data for more than six million patients. Source feeds include UCSF Health Epic/APeX extracts (Clarity/Caboodle) and the California Death Registry. These feeds are de-identified and loaded into institutional data warehouses, where they are harmonized into a single OMOP environment for analysis. OMOP organizes information into clinician-readable domains, including patient demographics, encounters (visits), mortality, diagnoses, medications, procedures, devices, laboratory and vital measurements, and other clinical observations. A standardized vocabulary layer (International Classification of Diseases (ICD) and Systematized Nomenclature of Medicine - Clinical Terms (SNOMED CT) for diagnoses, RxNorm for medications, LOINC for laboratory tests) ensures terminology consistency across sources (OHDSI, 2025).

### Knowledge graph

To place EHR findings in a biological and therapeutic context, MedCP queries SPOKE, a curated biomedical knowledge graph assembled from dozens of expert-maintained sources (e.g., ChEMBL, Genome-Wide Association Study (GWAS) Catalog, MeSH, Reactome) (Morris et al., 2023). SPOKE prioritizes provenance over scale: each edge derives from an identified source ontology or database with documented curation policies, rather than from automated extraction of unstructured literature, and source-level metadata are preserved at the edge level so that downstream queries can be filtered or weighted by provenance. The snapshot used here contains ~44.7 million nodes and ~132.7 million edges. Coverage spans molecular, genetic, and clinical concepts, with particularly deep representation of protein-level entities (~39.5 million proteins plus ~25 thousand related domains/families/complexes treated as protein-level concepts in our analyses), compounds (~2.43 million), genetic variants (~1.04 million), organisms (~0.42 million), and clinically salient concepts including diseases (~11.7 thousand), clinical laboratory tests (~59 thousand), pathways (~4.9 thousand), anatomy (~15.5 thousand), and cell types (~2.8 thousand). Relationships connect these layers: ~69.2 million protein–small-molecule links (endogenous metabolites and pharmaceuticals), ~4.0 million compound-gene regulatory associations (approximately half up-regulation and half down-regulation), ~1.7 million variant-to-gene mappings together with ~9.4 million gene-to-protein encoding links that enable traversal from sequence variation to protein products, and extensive gene-disease and compound-disease associations. SPOKE is rebuilt weekly from its constituent sources, so upstream content becomes queryable without re-indexing, re-embedding or redeployment. For reproducibility, all benchmark runs reported here were executed on a single day against a frozen build (September 2025).

### Performance benchmark

To evaluate the real-world application of the MedCP framework, 100 benchmarking questions were drafted by two of the authors—a medical doctor (GB) and a biomedical informatician (WG)—independently of, and prior to, any query execution. Question content was anchored to recurring clinical and research questions and to standard observational-epidemiology exemplars from the literature; the question set was finalized before the system was run, and no question was added, removed, or reworded after the benchmark was executed. Each question was assigned *a priori* to one of 10 complexity tiers, ranging from direct factual lookups to multi-step inferential reasoning tasks, based on four pre-defined criteria: minimum number of tool calls expected to answer it, depth of inferential reasoning required, whether the answer required cross-source integration (*EHR only, EHR primary* ± *KG optionally, EHR* + *KG*), and the statistical method expected to be used. Tier definitions and the per-question stratification criteria are provided in Table 2 and in Supplementary Table 1. For each question, we assessed GPT-5.5 and Claude Opus 4.8 connected through MedCP under two configurations: (i) with access to both the EHR database and the SPOKE biomedical KG, and (ii) with access to the EHR database alone, without the KG. This yielded 400 model-configuration runs (100 questions × 2 models × 2 configurations). A knowledge-graph-only configuration was not evaluated because most of the 100 benchmark questions require patient-level information (cohort sizes, time-to-event, laboratory values, treatment patterns) that SPOKE does not contain by design; running such a configuration would have produced structurally unanswerable questions rather than a fair performance comparison. Two domain experts (a physician and a bioinformatician) assessed each model's output. Responses were scored using a three-point rubric: 0 = incorrect or misleading answer; 1 = mostly correct but with minor deviations from assumptions or modest execution flaws not affecting the overall conclusion; 2 = fully correct, precise, and well-justified (see Supplementary Data 2, 3 for the grading rubrics). For analysis, the physician and bioinformatician ratings were averaged for each run, yielding ordered scores of 0, 0.5, 1, 1.5, or 2. No separate adjudicated overall score was present in the current analysis files. Both component ratings and their mean are reported in Supplementary Data 1.

**Table 2:** Benchmarking questions, rationale, and tiers.

Tier	Scope	Example question
1	Baseline size of a population or exposure.	How many patients received any statin in the past year?
2	Frequencies with one simple strata or a small, predefined set.	For bronchiolitis, select one supportive care medication and show how often it appears in pediatric charts.
3	Event rates after a new exposure using routine outcomes.	For new loop-diuretic users, what proportion has potassium < 3.5 mmol/L within 30 days?
4	Temporal trends and epoch comparisons.	What trends are observed in monthly incident lung cancer diagnoses over the last 3 years?
5	Concept ranking and biological contextualization.	Which antidepressant classes are most commonly used in older adults (≥65)?
6	Comorbidity profiling, condition co-occurrence, and biological linkage	Among adults with diagnosed hypertension, which comorbidities co-occur most frequently, and what shared biological pathways might link them?
7	Utilize KG-relatedness to propose clinically coherent sets and quantify them in the EHR.	Among the diseases biologically related to multiple sclerosis, which are most frequently diagnosed here?
8	Compare two groups defined by a mechanism in a temporal window.	In patients with melanoma starting checkpoint inhibitors, do pharmacological classes differ in the rate of autoimmune side effects within a 12-month window?
9	Within a single cohort, compare multiple clinically relevant outcomes across classes/regimens over a specified time window.	In combined oral contraceptives vs. progestin-only methods, what 12-month rates of venous thromboembolism and migraine codes are observed?
10	Most demanding descriptive comparisons (incident or transition designs) with clear windows.	Among dopamine-agonist side effects, which are more common in Parkinson's patients starting these drugs in our cohort within 6 months of prescription?

Session files were presented to both graders as a single unlabeled set, with no indication of the configuration from which each originated. As both rubrics require inspection of the executed queries, the configuration remained potentially inferable from the query trace.

**Figure 2:**
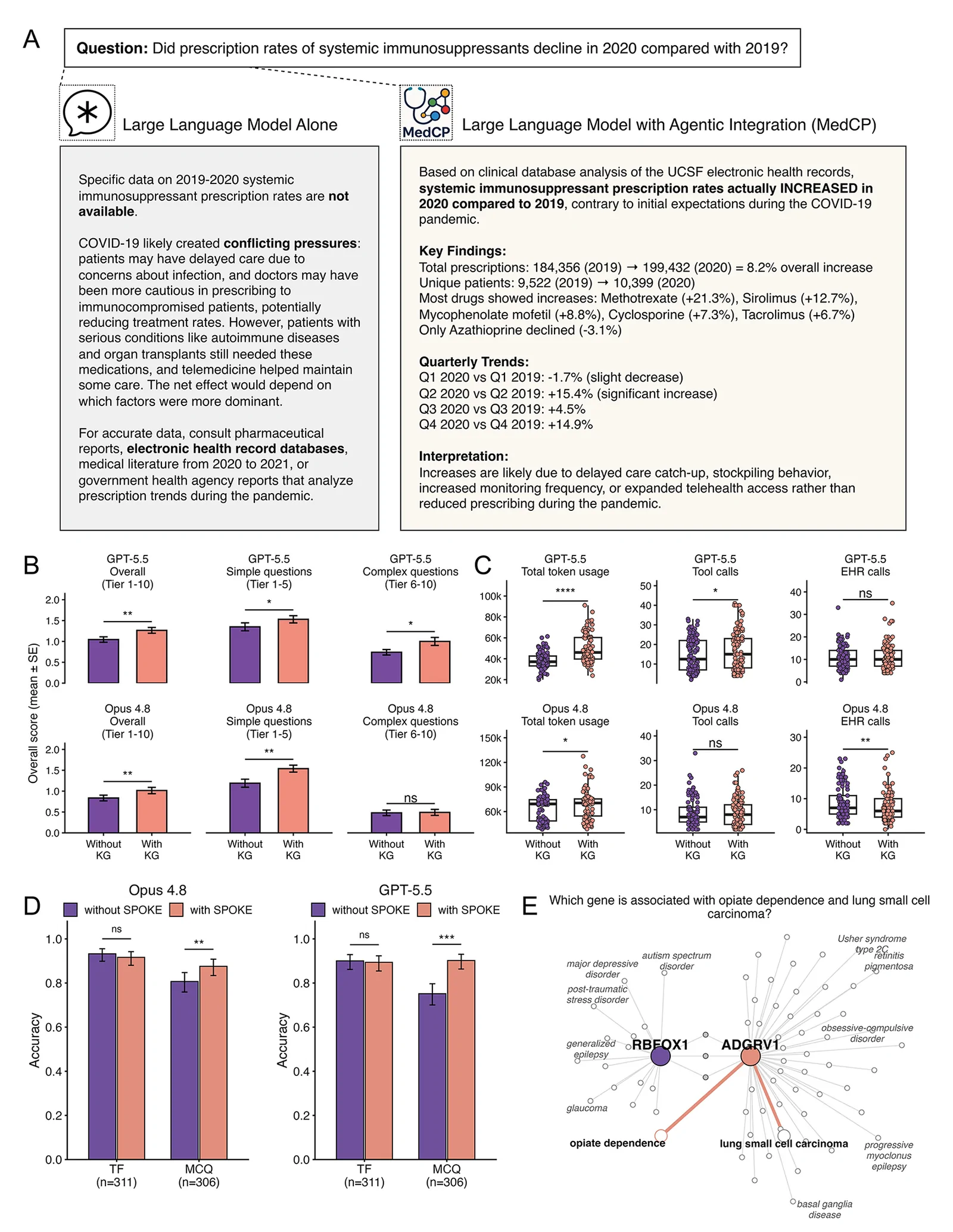
Benchmarking results of MedCP with and without the ability to query knowledge graphs. **(A)** Example question illustrating the added value of MedCP integration. A baseline large language model (LLM) alone produced a speculative narrative without data access, whereas the fully enabled MedCP (EHR + KG access) generated structured results based on UCSF electronic health records. **(B)** Mean overall benchmark scores (± SEM) for GPT-5.5 and Claude Opus 4.8 with (red) and without (purple) knowledge graph access across all 100 questions and stratified into lower-complexity (Tiers 1–5; *n* = 50) and higher-complexity (Tiers 6–10; *n* = 50) questions. Significance annotations compare paired with-KG vs. without-KG scores within each model and analysis scope using two-sided paired Wilcoxon signed-rank tests. Scores reflect the overall correctness and interpretability of responses: 0 = incorrect or misleading; 1 = mostly correct with minor deviations or execution flaws not substantially affecting the answer; 2 = fully correct, complete, and well-justified. **(C)** Per-question resource use for GPT-5.5 and Claude Opus 4.8 with and without knowledge graph access: total token usage, total tool calls (including EHR and knowledge graph queries), and EHR-specific calls. Points represent the 100 paired questions; boxplots show medians, interquartile ranges, and 1.5 × interquartile-range whiskers. Significance annotations compare paired with-KG vs. without-KG values within each model using two-sided paired *t*-tests. **(D)** Accuracy of BiomixQA answers generated without SPOKE and with SPOKE augmentation for true/false (TF; *n* = 311) and multiple-choice (MCQ; *n* = 306) questions, shown for Claude Opus 4.8 and GPT-5.5. Bars indicate mean accuracy and error bars indicate 95% Wilson confidence intervals. Significance annotations compare paired without-SPOKE vs. with-SPOKE performance within each question type using exact McNemar's tests. **(E)** Worked BiomixQA example illustrating how SPOKE graph grounding changes model selection for the question: “Which gene is associated with opiate dependence and lung small cell carcinoma?” The network shows SPOKE Gene-associates-Disease neighborhoods for RBFOX1 and ADGRV1. Disease nodes connected to each gene are shown in light gray, while highlighted orange edges indicate the disease associations supporting ADGRV1 as the SPOKE-grounded answer. Statistical significance: for **(B, C)** *p < 0.05, **p < 0.01, ***p < 0.001, and ****p < 0.0001; for **(D)** **p < 0.01 and ***p < 0.001; ns, not significant.

Knowledge-graph effects on the ordered response scores (0, 0.5, 1, 1.5, or 2) were tested separately for GPT-5.5 and Claude Opus 4.8 using proportional-odds ordinal logistic regression. We compared paired with-KG and without-KG runs across all 100 questions and within the prespecified Tiers 1–5 and Tiers 6–10 groups, using question-level clustering to account for repeated evaluation of the same questions. Odds ratios above 1 indicate higher scores with knowledge-graph access. The paired Wilcoxon signed-rank tests shown in Figure 2B served as a non-parametric sensitivity analysis.

We recorded secondary performance metrics, including execution time per question, total token use, total tool calls, knowledge graph calls, and EHR calls. Figure 2C compares total tokens, total tool calls, and EHR calls between configurations within each model using two-sided paired *t*-tests matched by question. Execution time was retained as a descriptive metric because timeout and retry loops produced unstable outliers. Statistical significance was assessed at *p* < 0.05.

Additionally, to test whether giving an LLM access to SPOKE via MedCP improves the factual accuracy on biomedical questions, we used BiomixQA, the question set publicly released with the KG-RAG framework (Soman et al., 2024), restricted to its gene–disease association items. The evaluation set comprised 617 questions: 311 true/false (TF) statements and 306 five-option multiple-choice questions (MCQ). Each MCQ presents a pair of diseases and five candidate genes, of which exactly one carries the queried association. Chance accuracy is 0.20 for MCQ and 0.50 for TF; because the TF items are imbalanced (197 true, 114 false), the appropriate TF reference is the majority-class baseline of 0.633. We tested two frontiers LLMs (Opus 4.8 and GPT 5.5), comparing performances with and without SPOKE (parametric memory alone, no tools, no web access). Accuracies are reported with 95% Wilson score intervals. The difference was measured via the exact (binomial) McNemar test on paired binary outcomes. applied within each model to compare the two arms, where *b* denotes items answered correctly only with SPOKE and *c* items answered correctly only without; the SPOKE contribution is the paired difference Δ = *(b–c)/n*. The same exact test was used to compare the two models within an arm. TF and MCQ were analyzed as pre-specified separate strata.

### Result interpretation and downstream reasoning

MedCP tools return structured data (CSV tables for SQL queries, JSON record lists for Cipher queries) rather than free-text answers; these structured outputs constitute intermediate artifacts that the orchestrating LLM then interprets to produce the final response. Depending on the question, this downstream reasoning may include simple summarization (e.g., counting and ranking), numerical synthesis (computing rates, ratios, or proportions from cohort counts), trend analysis (e.g., seasonal decomposition of monthly HbA1c measurements in question Q4.2), regression or survival analysis (e.g., propensity-score-matched Cox models in the replication framework), or visualization (Kaplan–Meier curves, bar charts). When such analyses exceed what can be expressed inside a single SQL/Cipher query, the LLM generates and executes Python code (using standard libraries such as pandas, statsmodels, lifelines, and matplotlib) on the structured intermediate output, rather than asking the database to perform the analysis. All generated code, the queries that produced its inputs, and the resulting figures and tables are preserved alongside the final answer in the run log, so that any post-query reasoning step is independently reviewable and reproducible. Representative end-to-end traces–prompt, intermediate JSON/CSV outputs, generated Python code, and final answer–are provided in the project repository.

### Case study: linking EHR epidemiology and knowledge graph biology

We used MedCP to extract the prevalence and co-occurrence of 11 common autoimmune diseases (e.g., psoriasis, rheumatoid arthritis, multiple sclerosis) and 11 neurological and neurodegenerative conditions (e.g., Alzheimer's disease, Parkinson's disease, unspecified dementia). Next, MedCP generated Cipher queries (to SPOKE) to quantify molecular similarity between disease pairs across seven dimensions: shared genes, genetic variants, clinical symptoms, anatomical localizations, therapeutic compounds, biological pathways, and curated disease–disease edges. A composite similarity network score was then calculated by dividing the observed overlap between each disease pair's neighborhoods by the overlap expected under independence:


$$\begin{array}{l} score_{AB}\;=\;\frac{\left|N_{A}\;\cap \;N_{B}\right|}{|N_{A}|\;\cdot \;|N_{B}|\;/\;N} \end{array}$$

Here, A and B denote the two diseases being compared; *N*_*A*_ and *N*_*B*_ are their respective sets of neighboring nodes; |*N*_*A*_ n N_*N*_*B*_| is the number of neighbors shared by both diseases; |*N*_*A*_| and |*N*_*B*_| are the numbers of neighbors in the two sets; *N* is the total number of nodes in the disease-related subgraph; and score_AB is the resulting normalized similarity (Leicht et al., 2006).

To test how network biology relates to epidemiology, MedCP correlated SPOKE similarity scores with observed EHR co-occurrence. The latter was adjusted for age, sex, follow-up duration and healthcare utilization measured as visits-days, and therefore represented as a Mantel-Haenszel adjusted Odds Ratio (aOR; Supplementary Figures 1A, B).

Associations were quantified with Spearman rank correlation, computed for all pairs and separately within immune-mediated pairs, within neurological pairs and across categories.

We also performed negative control analyses to assess the robustness of the EHR/network correlation, generating two sets of 1,000 permutations each on the knowledge graph: (a) matching for size, where each disease received a random neighborhood of its observed size drawn from the union of observed neighborhoods; (b) matching for neighborhood size and node degree (Strona et al., 2014), holding both each disease's neighborhood size and each node's connections, so perturbing the semantic structure of the network (Supplementary Figure 1C). Significance was assessed via a two-sided empirical *p*-value against the permutation distribution.

### Real-world study replication framework

To evaluate this framework's capacity to accelerate real-world evidence generation, we developed a standardized protocol for replicating published observational studies. This approach enables validation of prior findings across healthcare settings and testing of new hypotheses against the UCSF OMOP database through natural language interaction with MedCP. The protocol is implemented as a reusable skill within MedCP (“clinical-replication,” available in Supplementary Data 4) and structures the workflow into five sequential phases: (1) study deconstruction, extracting cohort definitions, exposures, outcomes, covariates, and statistical methods from the original publication; (2) OMOP concept mapping, leveraging hierarchical concept_ancestor relationships to translate clinical concepts into standardized vocabulary codes; (3) cohort extraction via optimized SQL queries with adaptive strategies for database limitations, including chunked extraction and database-level aggregation; (4) statistical analysis replicating the original methods, including survival analysis, propensity score methods, and power calculations; and (5) validation reporting with side-by-side comparisons, visual reproductions of key figures, and transparent documentation of limitations. Before data extraction, the protocol requires explicit specification of the reporting standard, primary estimand, time zero, eligibility and lookback windows, follow-up, censoring, competing risks, and checks for immortal-time bias. It also enforces reproducibility and privacy safeguards through aggregate-only reporting, audit trails of concept mappings and analytic choices, file-based storage of large query results, and mandatory reporting of event counts, person-time, model diagnostics, and minimum detectable effect estimates.

We applied this framework to studies published between 2019 and 2025 across multiple medical areas. Here, we discuss two exemplar settings with distinct characteristics: a study of GLP-1 receptor agonists (GLP-1RA) and dementia risk originally conducted on an international medical database (Anagnostakis et al., 2026), representing external validation across a different data source and patient population; and a safety study in multiple sclerosis originally performed on UCSF data, representing internal reproducibility and methodological consistency assessment (Cerono et al., 2026).

To show that MedCP capabilities transfer across OMOP-compliant databases, the same framework was applied to five original studies for demonstration purposes. They were conducted on the Medical Information Mart for Intensive Care (MIMIC) dataset distributed through the Eunomia sample-dataset manager, a conversion of the 100-patient MIMIC-IV demonstration cohort to the OMOP Common Data Model (Kallfelz et al., 2021; Johnson et al., 2023; DeFalco et al., 2025), which is a deployment distinct from the institutional instance used elsewhere in this work. Because that dataset is publicly redistributable, we also provide the configuration needed to connect the framework to it, so that MedCP can be tested without institutional data access. The research question as posed (prompt), the executed queries, the generated analysis code, the diagnostics and the final report are deposited for every study (see Data availability statement).

## Results

### Framework overview and user interaction workflow

MedCP connects LLMs to institutional data sources through a standardized interface enabling secure, schema-aware queries (Figure 1). Users interact conversationally with the LLM, which, in our case, decomposes clinical questions into parameterized SQL queries for the EHR database and Cipher queries for the SPOKE biomedical knowledge graph. The framework operates through read-only tools running within the health system network; queries execute locally under least-privileged roles, and only de-identified or aggregate results are returned. The UCSF Observational Medical Outcomes Partnership (OMOP) instance contains longitudinal records for over six million patients across standardized domains (demographics, diagnoses, medications, laboratory measurements), while SPOKE encompasses approximately 45 million nodes and 133 million edges representing molecular, genetic, and clinical relationships curated from dozens of biomedical sources (Morris et al., 2023; see Methods). At setup, users configure local endpoints and credentials for OMOP and SPOKE within MedCP. During use, the clinician or researcher poses a question in natural language. The LLM decomposes the question into sub-tasks, selects the appropriate MedCP tools, and synthesizes schema-aware, read-only SQL/Cipher queries. MedCP executes these queries locally, records audit metadata, and returns structured results–for example, cohort counts and timelines from OMOP or concept-to-concept paths from SPOKE. These queries run securely within the institutional network, returning only minimal, de-identified results while recording full provenance and execution logs. Outputs–tables, summaries, or figures–are delivered alongside the underlying queries, ensuring auditability and reproducibility while preserving data privacy.

### Knowledge graph access improves real-world research task performance and biomedical factual accuracy

To evaluate MedCP utility in real-world applications, we developed a set of 100 benchmarking clinical questions spanning multiple medical domains. These were constructed independently of MedCP and stratified in 10 tiers of growing complexity (Table 2, Supplementary Table 1); the answers were independently assessed and graded by domain experts, a clinician, and a bioinformatician (see Methods). Full per-question answers and grading are provided in Supplementary Data 1.

Across the 400 model-configuration runs, the great majority of generated queries were syntactically valid; we annotated a small number of formally invalid queries (e.g., undefined aliases, placeholder-only WHERE clauses, or prose without executable code) and observed no infinite loops or pathological over-nesting. Knowledge graph-enabled MedCP produced higher scores overall for both models (Figures 2A, B). For GPT-5.5, the mean score increased from 1.05 without the knowledge graph to 1.27 with it (ordinal OR 1.78, 95% CI 1.22–2.61, *p* = 0.0029); for Claude Opus 4.8, it increased from 0.84 to 1.02 (OR 1.50, 95% CI 1.09–2.05, *p* = 0.013).

Tier-stratified results differed by model. For GPT-5.5, knowledge graph access improved lower-tier scores (1.53 vs. 1.35; OR 1.73, 95% CI 1.02–2.92, *p* = 0.040) and showed a similar but non-significant tendency in Tiers 6–10 (1.00 vs. 0.74; OR 1.93, 95% CI 0.93–4.02, *p* = 0.079). For Claude Opus 4.8, the benefit was concentrated in Tiers 1–5 (1.54 vs. 1.19; OR 2.72, 95% CI 1.62–4.55, *p* < 0.001), with no difference in Tiers 6–10 (0.49 vs. 0.48; OR 0.98, 95% CI 0.54–1.78, *p* = 0.958). The tier-stratified estimates therefore support an overall knowledge-graph benefit, but do not show a uniform increase in benefit with question complexity (Figure 2B). Exploratively, we also analyzed the answers stratifying by the *a priori* source-requirement categories defined in Supplementary Table 1: EHR only (tiers 1–4; *n* = 40), EHR + optional KG (tiers 5 and 9; *n* = 20), and EHR + KG (tiers 6, 7, 8, and 10; *n* = 40). For GPT-5.5, the clearest gain appeared in EHR + KG questions (1.06 vs. 0.71; OR 2.63, 95% CI 1.16–5.97; *p* = 0.021), with smaller non-significant differences in EHR-only and EHR + optional KG questions. For Claude Opus 4.8, KG access improved EHR-only questions (1.55 vs. 1.29; OR 2.26, 95% CI 1.26–4.05; *p* = 0.006) and EHR + optional KG questions (0.95 vs. 0.48; OR 3.85, 95% CI 1.69–8.77; *p* = 0.001), while EHR + KG questions showed no significant improvement, further indicating that the effect of KG access was measurable, but model- and task-dependent. Scores were lower with knowledge graph access for 22 GPT-5.5 and 15 Claude Opus 4.8 questions; recurrent causes remained specific query-construction and ontology-handling errors rather than a general limitation of knowledge graph integration (see Supplementary Data 1).

Representative grading anchors illustrate the three-point rubric: GPT-5.5 with knowledge-graph access received a mean score of 2 for Q1.1 (a direct statin-use count, with both graders assigning 2), 1 for Q3.3 (the discontinuation estimate was useful but lacked a pre-index washout to establish incident SSRI use), and 0 for Q4.5 (data-truncation, cohort, and potassium-unit problems invalidated the requested temporal trend).

Questions 7.1 and 7.9 illustrated distinct changes in tool use. For Q7.1, KG-enabled runs used SPOKE-derived biological relatedness to define candidate diseases for EHR prevalence ranking, whereas EHR-only runs relied on manually selected or *ad hoc* disease sets. For Q7.9, KG-enabled runs used Attention-Deficit/Hyperactivity Disorder (ADHD)-related neurotransmitter pathways and pathway-aligned medications to structure the EHR utilization analysis, while EHR-only runs relied on manually specified medication/pathway groupings from parametric knowledge. Submaximal runs most often reflected workflow-level failures, including incomplete EHR/SQL temporal logic, nonspecific KG traversal, incomplete graph-to-OMOP mapping, or failure to synthesize clinical and mechanistic evidence; representative examples are provided in Supplementary Table 2.

Resource effects were also model-specific (Figure 2C). For GPT-5.5, knowledge graph access increased median token use (46,019 vs. 37,161; paired *p* < 0.0001) and total tool calls (15 vs. 12.5; *p* = 0.012) but not EHR calls (10 vs. 10; *p* = 0.566). For Claude Opus 4.8, token use increased slightly (70,434 vs. 69,317; *p* = 0.030), total tool calls were unchanged (8 vs. 7; *p* = 0.843), and EHR calls decreased (6 vs. 7; *p* = 0.0044). Although not shown in Figure 2C, median elapsed time was 396 vs. 592 s for GPT-5.5 (with vs. without the knowledge graph; paired *p* = 0.069) and 276 vs. 337 s for Claude Opus 4.8 (*p* = 0.021); these descriptive timings may be biased by API latency, network conditions, timeouts, and retries and should not be interpreted as intrinsic model speed. Thus, the score gains were achieved with modest, model-dependent resource tradeoffs rather than a uniform increase in database querying.

Finally, we evaluated whether grounding in SPOKE, accessed via MedCP, improves the factual accuracy of two frontier LLMs on biomedical questions, specifically gene-disease pairing from the BiomixQA benchmarking dataset, paralleling what has been done for testing SPOKE with a RAG infrastructure (Soman et al., 2024). Claude Opus 4.8 and GPT-5.5 each answered 311 true/false statements and 306 five-option multiple-choice questions (MCQ) twice: once from parametric memory alone, with no tools and no web access, and once with dedicated access to SPOKE via MedCP. Multiple-choice accuracy rose from 0.807 (95% CI 0.759–0.847) to 0.876 (0.834–0.908) for Opus 4.8, a paired gain of +6.9 percentage points (McNemar *p* = 0.0055), and from 0.752 (0.700–0.797) to 0.902 (0.863–0.930) for GPT-5.5, +15.0 points (*p* < 0.001), equivalent to a 35.6% and a 60.5% reduction in errors, respectively (Figure 2D). True/false accuracy did not change in either model (Opus 4.8 0.932 → 0.916, *p* = 0.53; GPT-5.5 0.900 → 0.894, *p* = 0.89), and both unaided TF accuracies already exceeded the 0.633 majority-class baseline by a wide margin. On identical multiple-choice items, GPT-5.5 was significantly less accurate than Opus 4.8 without the graph (0.752 vs. 0.807, *p* = 0.0076) but statistically indistinguishable from it with the graph (0.902 vs. 0.876, *p* = 0.17). Among multiple-choice items answered incorrectly without the graph, grounding recovered 37 of 59 for Opus 4.8 (62.7%) and 60 of 76 for GPT-5.5 (78.9%; Fisher *p* = 0.053); among items already correct, it reversed 16 of 247 (6.5%) and 14 of 230 (6.1%), an indistinguishable cost (Fisher OR = 0.94, *p* = 1.0). Question *MC0173* is representative of SPOKE utility (Figure 2E): the question asks which gene among *IL23R, HLA-DQB2, ADGRV1, DLEU7 and RBFOX1* is associated with opiate dependence and lung small cell carcinoma. Based solely on parametric memory, both models identified *RBFOX1*, a neuronal splicing regulator with a large neuropsychiatric and substance-use genetics literature support (O'Leary et al., 2022). The correct answer is *ADGRV1*. Given access to SPOKE, GPT-5.5 reached it by querying the graph, where it is represented as a gene-disease association (sourced from the GWAS catalog; Figure 2E).

### Molecular connectivity in the KG mirrors clinical disease co-occurrence in the EHR

**Figure 3:**
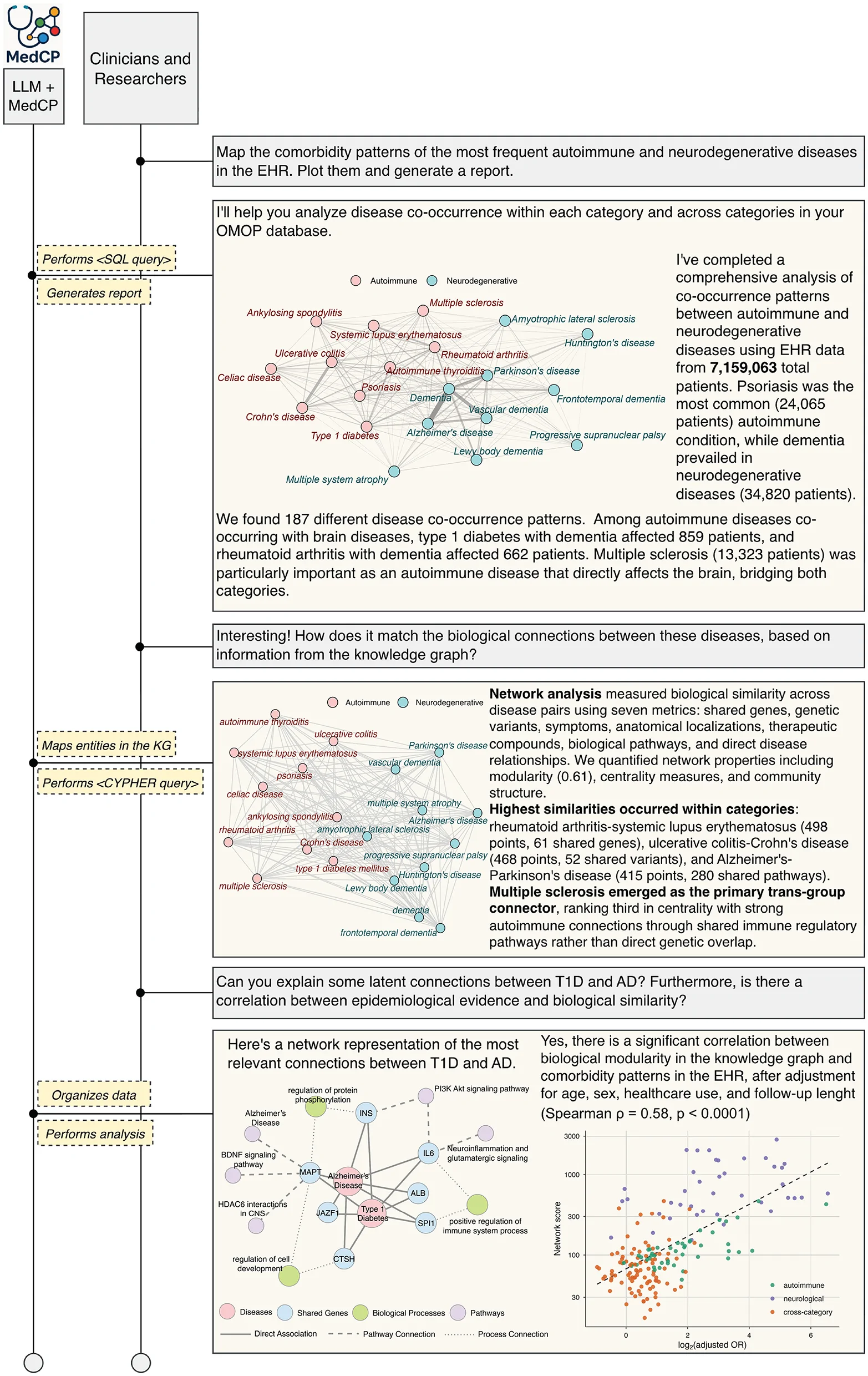
MedCP Integration of Clinical and Molecular Data Reveals Biological Basis of Disease Co-occurrence. Natural language dialogue demonstrating MedCP's integration of EHR and knowledge graph data. **(Top)** Analysis of 3,382,104 subjects with ≥1 recorded condition identifies 208 disease co-occurrence patterns between autoimmune (red nodes) and neurodegenerative diseases (blue nodes). **(Middle)** SPOKE biological network analysis reveals disease modularity (0.61) based on seven molecular dimensions. **(Bottom left)** Molecular subnetwork connecting type 1 diabetes and Alzheimer's disease shows seven shared genes and convergent biological processes, including neuroinflammation and CNS interactions involving HDAC6. **(Bottom right)** Biological similarity scores are associated with clinical co-occurrence frequencies measured as odds ratios (aOR) adjusted for age, sex, healthcare utilization and observation time (Spearman ρ = 0.58, *p* < 0.0001), suggesting that that molecular connectivity captures clinically meaningful disease relationships.

Network medicine holds that diseases sharing molecular determinants (genes, pathways, and interactome neighborhoods) tend to be phenotypically related and to co-occur in patients (Barabási et al., 2011; Menche et al., 2015). As an integrative case study, we used the MCP framework to explore, within a single agentic workflow, whether clinical co-occurrence in autoimmune and neurodegenerative diseases is consistent with this shared-biology hypothesis and could be used to generate mechanistic hypotheses from real-world data (Figure 3). Using natural language prompts, MedCP extracted disease prevalence, pairwise co-occurrence, and molecular similarity from the EHR and SPOKE, respectively. Among the UCSF study population, autoimmune conditions were highly prevalent, including psoriasis (24,065 patients), type 1 diabetes (23,329), and rheumatoid arthritis (19,126). Neurodegenerative diseases showed comparable prevalence, led by non-specified dementia (34,820 cases), Parkinson's disease (17,394), and Alzheimer's disease (13,532). MedCP identified 208 distinct disease co-occurrence patterns through automated EHR analysis, with substantial cross-category associations: type 1 diabetes co-occurred with dementia in 859 patients, and rheumatoid arthritis with dementia in 662 patients.

Parallel analysis of the SPOKE knowledge graph revealed that diseases cluster into biologically coherent modules based on shared genes, variants, pathways, and curated disease links. The most substantial molecular similarities occurred within disease categories: rheumatoid arthritis and systemic lupus erythematosus shared 61 genes and 317 biological pathways, while Alzheimer's and Parkinson's disease converged on 280 shared molecular nodes. Multiple sclerosis emerged as a key bridge between autoimmune and neurodegenerative categories, ranking third in network centrality, with substantial connectivity to both disease clusters through shared genes, pathways, and immune regulatory mechanisms (Figure 3).

Detailed examination of specific cross-category connections revealed hypothesis-generating, mechanistic substrates. The type 1 diabetes–Alzheimer's disease association, for instance, converged on seven shared genes (including IL6, INS, and MAPT) and multiple biological processes spanning BDNF signaling, neuroinflammation, PI3K-Akt pathway activation, and immune system regulation. These molecular connections suggest candidate mechanisms underlying observed clinical associations that would be difficult to identify through either data source alone.

After adjustment for age, sex, length of follow-up and healthcare utilization, clinical co-occurrence across was concordant with network molecular similarity in the knowledge graph (Spearman ρ = 0.58, *p* < 0.0001; Figure 3). The association was stronger among the immune-mediated pairs (ρ = 0.61, *p* < 0.0001), than among the neurological pairs (ρ = 0.37). The global correlation was robust to sensitivity analysis under degree-preserving permutations of the knowledge graph (Supplementary Figure 1).

This result suggests that KG-derived biological relationships capture clinically meaningful biological connections. Overall, this case study shows how MedCP can translate real-world clinical observations into mechanistic hypotheses for follow-up, in a setting where the underlying biological relationships are already documented.

### Replication of real-world studies via MedCP demonstrates accelerated evidence generation

**Figure 4:**
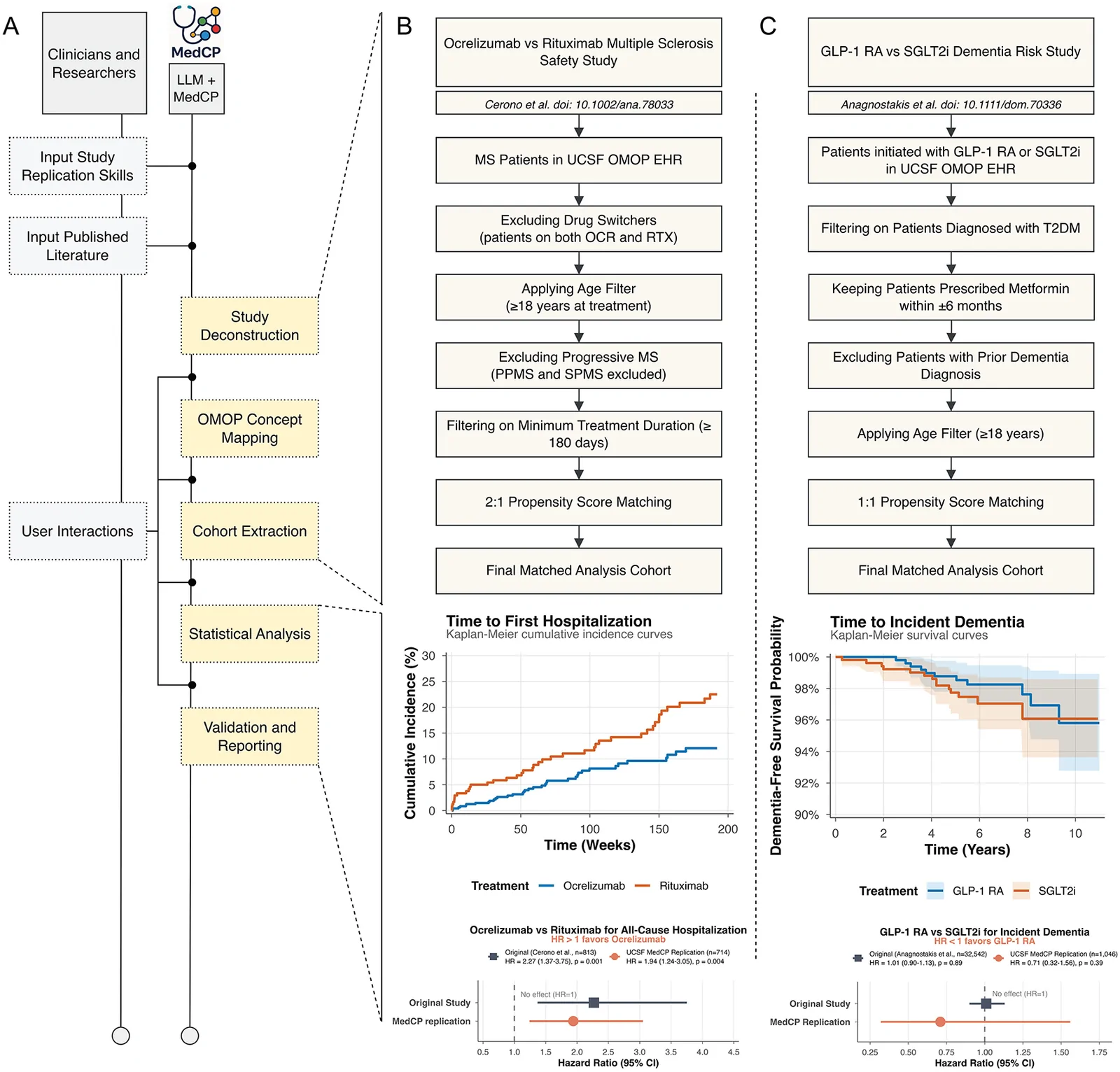
Performing and Replicating Real-World Studies with MedCP. **(A)** Five-phase replication workflow: study deconstruction extracts cohort definitions, exposures, outcomes, and statistical methods from published papers; OMOP concept mapping translates clinical terms to standardized vocabulary codes using hierarchical relationships; cohort extraction generates SQL queries with adaptive chunking for large populations; statistical analysis replicates original methods including survival analysis and propensity score matching; a report on reproducibility shows side-by-side comparisons with original findings. **(B)** Internal reproducibility example: Ocrelizumab vs. rituximab safety in multiple sclerosis. The original UCSF CDW study compared with the current OMOP instance replication. Hazard ratios for all-cause hospitalization with 95% confidence intervals demonstrate directional consistency. **(C)** External pipeline feasibility example: GLP-1 receptor agonist vs. SGLT2 inhibitor and dementia risk. Original study (Anagnostakis et al., 2026; TriNetX database, *n* = 32,542 matched pairs) compared with UCSF OMOP replication (*n* = 1,046 matched pairs). Kaplan–Meier curves and forest plot showing hazard ratios with 95% confidence intervals.

To demonstrate MedCP's utility in accelerating real-world evidence generation, we applied a standardized protocol to systematically replicate select observational studies published between 2019 and 2025 across several medical domains. As detailed in the Methods section, this protocol implements a five-phase systematic workflow encompassing study deconstruction, OMOP concept mapping using hierarchical vocabulary relationships, optimized cohort extraction with adaptive database strategies, statistical analysis replicating original methods, and comprehensive validation reporting with transparent limitation documentation (Figure 4, Supplementary Data 4). The complete replication workflow takes a total active work of less than 1 h, representing substantial acceleration compared to the weeks or months typically required for traditional real-world evidence studies.

Figure 4 presents two exemplar studies representing distinct feasibility testing scenarios. The first, reporting higher hospitalization rates in rituximab-treated MS patients (compared to ocrelizumab), was recently published by our group and was based on UCSF data; the second examined GLP-1 receptor agonist vs. SGLT2 inhibitor associations with incident dementia in type 2 diabetes, originally conducted by (Anagnostakis et al. 2026) on the TriNetX multi-site database. The two studies test different aspects of the workflow: the first, is replicated here against the OMOP instance and tests reproducibility across data models within one institution; the second, conducted elsewhere on a multi-site database, tests whether an external protocol can be executed end to end against a local cohort.

The ocrelizumab vs. rituximab in MS safety replication (Figure 4B) focused on all-cause hospitalization as the primary outcome, and hypogammaglobulinemia (IgG < 565 milligrams per deciliter) as the secondary outcome. We examined 714 patients with relapsing-remitting MS who received continuous therapy with either ocrelizumab (475 patients) or rituximab (239 patients), using a 2:1 propensity score-matching strategy similar to the original study design. After excluding patients who switched between the two anti-CD20 monoclonal antibodies during the observation period and those with progressive multiple sclerosis subtypes (primary progressive or secondary progressive), propensity score matching was performed on age, sex, disease duration, prior disease-modifying therapy exposure, and baseline comorbidities, including hypertension, diabetes, heart failure, chronic obstructive pulmonary disease, and smoking status. The UCSF replication yielded a higher hazard ratio of all-cause hospitalization for rituximab-treated patients (HR = 1.94, 95% C.I. 1.24–3.05, *p* = 0.0039), showing magnitude and directional consistency with the original study (HR = 2.27, 95% C.I 1.37–3.75, *p* = 0.001) in 813 patients. The hospitalization rates per 100 person-years were also comparable between studies, with the original reporting 3.47 for ocrelizumab and 8.01 for rituximab, while the replication observed 3.69 and 7.09, respectively, representing close alignment in absolute risk measures. For hypogammaglobulinemia, the replication HR of 1.85 (95% C.I. 0.67–5.10, *p* = 0.24) showed directional consistency with the original HR of 2.72 (95% C.I. 1.18–6.29, *p* = 0.003), but did not reach statistical significance. This attenuation likely reflects differences in data architecture between the Clinical Data Warehouse used in the original study—which provided direct access to all laboratory values—and the OMOP data model used in our replication, where laboratory results undergo vocabulary standardization that may incompletely capture specialty panels or non-standard local codes, reducing both event counts and statistical power.

For the GLP-1 RA vs. SGLT2i dementia risk replication (Figure 4C), we identified 1,372 eligible patients in the UCSF OMOP database who initiated either GLP-1 receptor agonists (773 patients) or SGLT2 inhibitors (599 patients) between April 2013 and December 2019, had documented type 2 diabetes mellitus, were prescribed metformin within 6 months of index medication initiation, had no prior dementia diagnosis, and were at least 18 years of age at treatment initiation. Following one-to-one propensity score matching using a nearest-neighbor algorithm with caliper of 0.1 standard deviations on age, sex, baseline comorbidities (hypertension, heart failure, obesity, chronic kidney disease, depression, anxiety, atrial fibrillation, ischemic heart disease, cerebrovascular disease, hyperlipidemia), and available laboratory values, the final matched analysis cohort comprised 1,046 patients (523 matched pairs). Over a median follow-up of 5.9 years, 25 incident dementia events occurred (11 in the GLP-1 receptor agonist group and 14 in the SGLT2 inhibitor group), yielding incidence rates of 3.2 per 1,000 person-years for GLP-1 receptor agonists and 4.4 per 1,000 person-years for SGLT2 inhibitors. The hazard ratio was 0.71 (95% C.I. 0.32–1.56, *p* = 0.39), suggesting a trend toward lower dementia risk with GLP-1 receptor agonist use that did not reach statistical significance. This differed in direction from the original study by Anagnostakis et al. (2026), which reported a null finding with hazard ratio of 1.01 (95% C.I. 0.90–1.13, *p* = 0.89) in 16,271 matched pairs with 1,153 total dementia events over median follow-up of 6.3 years. The original design was replicated end to end in the UCSF OMOP instance, covering cohort identification, application of all eligibility criteria, propensity score matching, and survival analysis. The resulting estimate, however, rests on a cohort comprising 3.2% of the original sample, and its confidence interval is too wide to distinguish a null effect from a clinically meaningful one. The replication is therefore best read as compatible with the original null finding rather than as an independent confirmation of it.

Additional examples of replications on real EHR data across pharmacovigilance, comparative effectiveness, and risk factor studies are documented in our repository, validating the framework's robustness across diverse observational research methodologies, along with five original studies conducted on a 100-patients sample of the MIMIC-IV dataset adapted to the OMOP data model (Supplementary Figure 2), that demonstrate MedCP translability to other OMOP instances.

## Discussion

In this work, we demonstrate how an agentic framework applying the Model Context Protocol to a large institutional EHR repository, together with a curated biomedical knowledge graph, can broaden access to, and accelerate, real-world biomedical evidence generation spanning clinical phenotypes and molecular pathways through conversational interaction, while retaining transparency for audition and experts' supervision.

Our results indicate that MCP provides a robust and scalable way to connect LLMs with heterogeneous biomedical resources. Its standardized interface enables new data sources to be added without redesigning retrieval pipelines, overcoming the rigidity that characterizes many RAG implementations (Ng et al., 2025). Crucially, every query produces explicit SQL or Cipher statements available for review and reporting, turning natural language interactions into fully documented computational steps.

Grounding LLM reasoning in curated data improved factual reliability. Across both evaluated models, adding the curated knowledge-graph layer improved scores overall compared with an EHR-only configuration, although tier-specific effects differed, echoing evidence that KG augmentation enhances accuracy and reduces unsupported claims compared with parametric models (Su et al., 2025). Conceptually, the KG functions as an evidential filter: curated molecular relationships constrain and direct the analysis toward biologically grounded hypotheses, rather than allowing the model to search clinical data guided only by parametric assumptions. This architectural grounding helps limiting hallucination, a relevant concern in biomedical LLM applications (Omar et al., 2025). The disease co-occurrence analysis illustrates this value concretely. By linking observed clinical co-occurrence patterns to molecular similarity through shared genes, pathways, and protein interactions, MedCP identified multiple sclerosis as a bridge between autoimmune and neurodegenerative disease cluster, an observation aligned with its established dual pathophysiology involving both immune-mediated demyelination and progressive neurodegeneration (Hauser and Oksenberg, 2006).

Overall, the benchmark outputs help clarify what the knowledge graph contributed operationally. SPOKE was most useful when a question required the agent to define an upstream analytic object before querying the EHR, such as a biologically related disease set or a pathway-aligned medication class. In Q7.1, graph-derived disease relationships provided an explicit candidate set for downstream EHR prevalence estimation; in the EHR-only runs, this set was instead defined through broad manual categories such as autoimmune or demyelinating disease. In Q7.9, KG-enabled runs used ADHD-related neurotransmitter pathways to structure medication-use analyses. These examples show that graph access can make biological assumptions more explicit and guide toward better evidence generation. The BiomixQA experiment provided an orthogonal validation of this grounding effect outside the EHR workflow: SPOKE access improved multiple-choice gene-disease question answering, consistent with prior SPOKE-based KG-RAG results (Soman et al., 2024), while showing that the same knowledge source can be exposed through an MCP tool interface rather than a retrieval-only architecture.

The benchmark also showed that knowledge-graph benefit and tool use were model-dependent. GPT-5.5 improved significantly overall and in Tiers 1–5, with a directionally similar higher-tier gain, whereas Claude Opus 48's benefit was concentrated in Tiers 1–5. These tier-specific differences are descriptive and should not be interpreted as a formal between-model comparison. Their execution profiles also differed: GPT-5.5 added total tool calls while maintaining similar EHR-call counts with knowledge-graph access, whereas Claude Opus 4.8 maintained total calls but made fewer EHR calls, suggesting deployment-specific tendencies to layer knowledge-graph retrieval onto EHR querying rather than substitute it.

Submaximal runs clustered around recurring workflow failures documented in Supplementary Table 2. At the EHR/SQL layer, common issues included temporal misalignment, inadequate washout or follow-up logic, denominator errors, and exposure or outcome misclassification. At the knowledge-graph layer, failures included incomplete traversal, nonspecific entity retrieval, and incomplete transfer of graph-derived candidates into OMOP cohorts. Multi-tool questions also exposed a synthesis step in which the clinical or mechanistic component could be completed without fully connecting the two. These patterns motivate concept-set validation, temporal-cohort templates, denominator checks, mapping-coverage reports, uncertainty reporting, and resumable execution under expert review.

MCP's direct query architecture offers distinct advantages over RAG approaches, including our previous work on KG-optimized prompt generation (Matsumoto et al., 2024; Soman et al., 2024). While RAG and KG-RAG systems effectively ground LLM responses in external knowledge, they require substantial infrastructure (vector databases, embedding models, and retrieval pipelines) that users must configure and maintain, and their embedded representations become stale as source data evolves unless periodically re-vectorized. MedCP, using the MCP system, circumvents these limitations through an agentic architecture where the LLM reasons about query strategy, invokes database tools, and iteratively refines results based on returned data. This architecture requires no local infrastructure beyond network access, no preprocessing or vectorization of source material, and ensures results always reflect the current database state at query time. Furthermore, native query languages support precise filtering, complex aggregations, temporal logic, and graph traversals that semantic similarity retrieval cannot express–capabilities essential for multilayered clinical questions. An empirical comparison of MCP- and RAG-augmented LLMs on clinical-guideline-derived questions found that both approaches improved accuracy over unaugmented models, and did so consistently across three LLM families (Zhao et al., 2026). The two architectures therefore differ less in accuracy than in scope: MCP operates on structured, queryable databases rather than arbitrary text corpora, where RAG excels; for biomedical research applications leveraging standardized resources, direct query access may offer advantages in precision, currency, and accessibility.

Concerns about LLM accuracy, reproducibility, and transparency have motivated extensive work on model-level interpretability (e.g., feature attribution and attention analysis); these techniques, however, are designed for supervised models with a single output and do not have a clean analog in agentic, multi-turn workflows where a single answer emerges from many tool calls and intermediate reasoning steps (Mesinovic et al., 2025). Rather than attempting parametric interpretability of the underlying LLM, MedCP provides procedural auditability: every clinical or biological claim returned to the user is traceable to one or more explicit, human-readable database queries (SQL or Cipher). Domain experts can re-run any query, modify it, and verify that the cited numbers reflect the current state of the database. This trace-level transparency does not explain *why* the model chose a given query, but it does answer the question that matters most for clinical research integrity, namely *what was computed and against which data*. We view procedural auditability and parametric interpretability as complementary rather than substitutable goals; in safety-critical evidence-generation settings, auditability of the data path is arguably the more directly actionable property.

Several recent efforts have explored agentic or protocol-driven access to clinical data and biomedical knowledge, including MCP-based connectors to FHIR resources (Ehtesham et al., 2025), hospital-EHR retrieval evaluations (Masayoshi et al., 2025), KG-augmented question-answering frameworks and agents (Feng et al., 2025; Su et al., 2025; Xiong et al., 2025), and EHR-grounded medical agent benchmarks (Jiang et al., 2025). As Supplementary Table 3 shows, these systems occupy distinct niches. The KG frameworks, including our own earlier KG-RAG (Soman et al., 2024), KRAGEN (Matsumoto et al., 2024), and more recent agents built on PrimeKG or domain-specific graphs (Feng et al., 2025; Su et al., 2025), ground language models in curated biomedical knowledge for question answering, but do not reach institutional patient records; the FHIR-based systems (Ehtesham et al., 2025; Jiang et al., 2025) reach real or synthetic clinical records but not a molecular knowledge graph. Compared with these efforts, the deployment we report here through MedCP brings together three properties that, to our knowledge, no prior published clinical-AI application has reported jointly: (i) institutional-scale OMOP EHR access through a standardized, schema-aware MCP layer, under institutional security controls; (ii) integration with a large, curated biomedical knowledge graph in the same agentic workflow, and (iii) end-to-end demonstration on real-world evidence generation tasks. MedCP builds on OMOP rather than FHIR because its target is cohort-level observational evidence: FHIR standardizes real-time exchange of individual records, OMOP harmonizes data for population-scale analysis, and the two are complementary layers.

Likewise, efforts like MedCP can complement the OHDSI analytic ecosystem for OMOP data. ATLAS and CIRCE for cohort definition and the HADES packages for population-level estimation and prediction (Hripcsak et al., 2015; The Book of OHDSI, 2021) remain the community standard for validated, reproducible observational analysis. These are programmatic, deterministic tools; MedCP instead provides a natural-language interface that accompanies query construction and analysis across both the EHR and a knowledge graph. This cross-source, interactive dimension lies outside the scope of the OMOP-only OHDSI tools.

Our study replication framework relates to emerging LLM-driven research paradigms that keep information traceable under human oversight. For example, Ifargan et al. (2025) recently reported on a “data-to-paper” automation platform guiding LLM agents through complete research workflows, while maintaining information traceability. Applying this paradigm to institutional clinical data can assist researchers in specifying and executing real-world studies through conversational interaction, as illustrated by the case studies above. Similarly, this approach extends to target trial emulation, an increasingly influential methodology for causal inference when randomized trials are infeasible (Hernán et al., 2022). Target trial emulation requires precise specification of eligibility criteria, treatment strategies, outcome definitions, and causal contrasts, which a framework like MedCP can help translate into executable queries. A persistent challenge in this methodology has been the gap between clinical protocol specification and computational implementation, where translation errors may remain undetected until late-stage analysis (Hernán, 2021). By allowing iterative refinement of cohort definitions and analytical assumptions (via user-agent interaction), such approaches reduce this specification-implementation gap while maintaining methodological transparency.

Several limitations warrant consideration. First, evaluation was conducted at a single academic medical center; the UCSF patient population may not generalize to community practice, and variables such as pharmacy claims and patient-reported outcomes were incompletely captured in the local OMOP instance. These constraints are common to all single-site observational studies, and MedCP's standardized architecture facilitates future multi-site validation through consistent query deployment across OMOP-compliant databases.

Second, although the framework architecture is model-agnostic, the empirical results here (benchmark and case studies) are deployment-specific. Model choice was restricted to the options available through the secure UCSF endpoint.

Third, the results of MedCP queries depend on underlying data quality. For EHR, as in any other real-world data analysis, possible issues include coding errors, missing values, and surveillance bias. The knowledge graph, instead, can miss biomedical relationship established after the last ingest of a source database. Our framework mitigates interpretive risk by exposing both failure modes in the query audit trail, which allows expert review.

Fourth, current language models impose context-window constraints on large result sets; queries returning datasets exceeding approximately one megabyte may trigger context overflow. Our framework addresses this by generating executable code that researchers can run locally on exported data, preserving analytical capability while offloading computation to appropriate environments.

Importantly, as any other LLM-driven system to date, MedCP is an example of an agentic-AI biomedical research harness that assists human experts to accelerate hypothesis generation and testing. In fact, although MedCP lowers technical barriers to data access, appropriate interpretation still requires foundational epidemiological and statistical expertise. The architecture supports such expert human oversight by exposing every query and analytical step for review.

Future development should integrate additional biomedical resources, including genomic repositories, imaging archives, clinical trial registries (Acosta et al., 2022), as well as the primary literature. Indeed, agentic systems that construct knowledge graphs from the literature and reason over them with explicit provenance to primary evidence are advancing rapidly (Sanda et al., 2026; Wang et al., 2026). Nonetheless, while architecturally simple (a bibliographic server can be attached to the same client as an additional endpoint), the semantic entities resolution and relational modeling require an additional layer of expert supervision and dedicated testing (Gao S. et al., 2025).

Finally, multi-institutional deployment of MedCP and similar tools across federated OMOP networks would establish reliable performance in routine institutional use while enabling cross-population generalization without centralizing sensitive data.

## Conclusions

In conclusion, this work demonstrates that applying the Model Context Protocol to a real institutional EHR and a curated biomedical knowledge graph can accelerate hypothesis generation and real-world evidence studies for biomedical researchers. In the deployment reported here, complex data queries are turned into interactive conversations that compress workflows traditionally requiring weeks into sessions lasting minutes to hours, with every generated query remaining visible for expert review, every analytical step logged for reproducibility, and interpretation kept under domain-expert supervision. This kind of agentic pipeline augments rather than replaces experts' judgment: it aids in the technical translation between clinical questions and structured data, while researchers retain authority over study design and scientific inference.

## Data Availability

The MedCP GitHub repository (https://github.com/BaranziniLab/MedCP) includes the source code, MCP server implementation, Claude Desktop extension, benchmarking questions, benchmark scoring sheets (including submaximal and failed cases), case study prompts, examples of replicated study workflows, synthetic dataset acces instructions, generated reports, and intermediate structured outputs, including SQL/Cypher query outputs in JSON or CSV format. These materials are intended to document how MedCP decomposes user questions, retrieves structured data from EHR and KG sources, and synthesizes final responses, including examples in which additional post-query reasoning, tabulation, visualization, or report generation was required. Detailed tool documentation is available at https://baranzinilab.github.io/MedCP/.

## Supplementary material

Supplementary Table 1 Criteria used to assign MedCP benchmark questions to complexity tiers (expected tool calls, reasoning depth, cross-source integration, statistical method).

Supplementary Table 2 Model-agnostic failure modes across EHR/SQL, knowledge-graph, and combined workflows in submaximal-score responses to the 100-question benchmark, with cross-model examples and safeguards.

Supplementary Table 3 Comparison of MedCP with related clinical and biomedical agent frameworks.

Supplementary Data 1 Grading results for GPT-5.5 and Claude Opus 4.8 with and without knowledge graph access across the 100-question benchmark.

Supplementary Data 2 Grading rubric for clinician evaluators assessing clinical correctness, interpretability, and data-groundedness of MedCP responses.

Supplementary Data 3 Grading rubric for bioinformatician evaluators auditing SQL and Cipher query correctness and efficiency in MedCP tool calls.

Supplementary Data 4 MedCP clinical replication skill framework for reproducing peer-reviewed clinical studies, including data workflow protocols, statistical analysis guidelines, reporting checklists, and replication report generation.

Supplementary Figure 1 Case study cohort structure and negative control analysis. **(A)** Age at first recorded diagnosis, one weighted density per disease. **(B)** Distribution of the full observation-period population (gray) and of disease cases (red) across the stratification axes. **(C)** Null distributions of the pooled Spearman correlation under two randomizations of the knowledge graph, 1,000 permutations each; the red line marks the observed value (ρ = 0.580).

Supplementary Figure 2 Overview of five demonstration studies conducted via MedCP on MIMIC-IV Eunomia OMOP dataset (*N* patients = 100). Each row **(A–E)** is one study: the card gives the verbatim request put to the agent, the three-step procedure it executed and the headline output; **(A)** Atrial fibrillation and anticoagulation coverage (*n* = 23): in-hospital mortality by anticoagulant exposure (heparin 15, none 8), and the odds ratio for death with 95% CI (Fisher exact test; dashed line, OR = 1). **(B)** Blood lactate and in-hospital mortality (*n* = 83, 15 deaths): peak lactate by outcome on a log_10_ axis (Mann–Whitney *U*), and Kaplan–Meier survival by lactate tertile to a 28-day in-hospital landmark (log-rank). **(C)** Polypharmacy and QT-prolonging drug burden (*n* = 77 on ≥5 named drugs): name-resolution coverage of drug-exposure rows — the unresolved raw-NDC stratum holds the 83 SPOKE-flagged QT-prolonging agents, leaving QT burden non-estimable — and named medications per patient (dashed line, median). **(D)** Opioid exposure and respiratory parameters (38 exposed, 62 unexposed): nadir SpO_2_ against per-patient reading count with LOESS trend (Spearman ρ), which exposes the effect as surveillance bias, and nadir SpO_2_ by exposure group (Mann–Whitney *U*). **(E)** Coded comorbidity and shared-gene networks: comorbidity network of the 19 ICD-derived diseases resolved to SPOKE Disease nodes. For each study the verbatim launching prompt, the complete MCP query log, the analysis and figure code, the extracted EHR and knowledge-graph artifacts, and the full HTML report are deposited in the MedCP repository.

## References

[B1] Acosta J. N. Falcone G. J. Rajpurkar P. Topol E. J. (2022). Multimodal biomedical AI. Nat. Med. 28, 1773–1784. doi: 10.1038/s41591-022-01981-2 36109635

[B2] Anagnostakis F. Kokkorakis M. Nagarajan S. Anastasiou G. Mantzoros C. S. (2026). Comparative effectiveness of sodium-glucose cotransporter-2 inhibitors and glucagon-like peptide-1 receptor agonists for incident dementia: a retrospective multicohort study. Diabetes Obes. Metab. 28, 1443–1452. doi: 10.1111/dom.70336 41309383 PMC12803637

[B3] Barabási A.-L. Gulbahce N. Loscalzo J. (2011). Network medicine: a network-based approach to human disease. Nat. Rev. Genet. 12, 56–68. doi: 10.1038/nrg2918 21164525 PMC3140052

[B4] Bartlett V. L. Dhruva S. S. Shah N. D. Ryan P. Ross J. S. (2019). Feasibility of using real-world data to replicate clinical trial evidence. JAMA Netw. Open 2:e1912869. doi: 10.1001/jamanetworkopen.2019.12869 31596493 PMC6802419

[B5] Cerono G. Cree B. A. C. Hauser S. L. Baranzini S. E. (2026). Comparative safety profiles of ocrelizumab and rituximab in multiple sclerosis treatment using real-world evidence. Ann. Neurol. 99, 248–260. doi: 10.1002/ana.78033 40919837 PMC12946588

[B6] Choi J. Y. Yoo T. K. (2026). Transforming clinical medicine with multimodal artificial intelligence, agentic systems, and the model-context protocol: a perspective on future directions. Discov Health Syst. 5:6. doi: 10.1007/s44250-026-00343-w

[B7] Davenport T. Kalakota R. (2019). The potential for artificial intelligence in healthcare. Future Healthc. J. 6, 94–98. doi: 10.7861/futurehosp.6-2-94 31363513 PMC6616181

[B8] DeFalco F. Schuemie M. Sena A. Adulyanukosol N. Liu S. Black A. (2025). Eunomia: Standard Dataset Manager for Observational Medical Outcomes Partnership Common Data Model Sample Datasets. Eunomia. Available online at: https://ohdsi.github.io/Eunomia/ (Accessed July 30, 2026).

[B9] Ehtesham A. Singh A. Kumar S. (2025). “Enhancing clinical decision support and EHR insights through LLMs and the model context protocol: an open-source MCP-FHIR framework,” in Proceedings of the 2025 IEEE World AI IoT Congress (AIIoT), Seattle, WA, USA (IEEE: New York, NY). doi: 10.1109/AIIoT65859.2025.11105280

[B10] Feng Y. Zhou L. Ma C. Zheng Y. He R. Li Y. (2025). Knowledge graph–based thought: a knowledge graph–enhanced LLM framework for pan-cancer question answering. GigaScience 14:giae082. doi: 10.1093/gigascience/giae082 39775838 PMC11702363

[B11] Gao S. Yu K. Yang Y. Yu S. Shi C. Wang X. . (2025). Large language model powered knowledge graph construction for mental health exploration. Nat. Commun. 16:7526. doi: 10.1038/s41467-025-62781-z PMC12350947 40804250

[B12] Gao Y. Li R. Croxford E. Caskey J. Patterson B. W. Churpek M. . (2025). Leveraging medical knowledge graphs into large language models for diagnosis prediction: design and application study. JMIR AI 4:e58670. doi: 10.2196/58670 39993309 PMC11894347

[B13] Hauser S. L. Oksenberg J. R. (2006). The neurobiology of multiple sclerosis: genes, inflammation, and neurodegeneration. Neuron 52, 61–76. doi: 10.1016/j.neuron.2006.09.011 17015227

[B14] Hernán M. A. (2021). Methods of public health research—strengthening causal inference from observational data. N. Engl. J. Med. 385, 1345–1348. doi: 10.1056/NEJMp2113319 34596980

[B15] Hernán M. A. Wang W. Leaf D. E. (2022). Target trial emulation. JAMA 328:2446. doi: 10.1001/jama.2022.21383 36508210

[B16] Hripcsak G. Duke J. D. Shah N. H. Reich C. G. Huser V. Schuemie M. J. . (2015). Observational health data sciences and informatics (OHDSI): opportunities for observational researchers. Stud. Health Technol. Inform. 216, 574–578. doi: 10.3233/978-1-61499-564-7-574 26262116 PMC4815923

[B17] Ifargan T. Hafner L. Kern M. Alcalay O. Kishony R. (2025). Autonomous LLM-driven research—from data to human-verifiable research papers. NEJM AI 2:AIoa2400555. doi: 10.1056/AIoa2400555

[B18] Jiang Y. Black K. C. Geng G. Park D. Zou J. Ng A. Y. . (2025). MedAgentBench: a virtual EHR environment to benchmark medical LLM agents. NEJM AI 2:AIdbp2500144. doi: 10.1056/AIdbp2500144

[B19] Johnson A. E. W. Bulgarelli L. Shen L. Gayles A. Shammout A. Horng S. . (2023). MIMIC-IV, a freely accessible electronic health record dataset. Sci. Data 10:1. doi: 10.1038/s41597-022-01899-x PMC9810617 36596836

[B20] Kallfelz M. Tsvetkova A. Pollard T. Kwong M. Lipori G. Huser V. . (2021). MIMIC-IV Demo Data in the OMOP Common Data Model. PhysioNet.

[B21] Kim Y. Jeong H. Chen S. Li S. S. Lu M. Alhamoud K. . (2025). Medical Hallucinations in Foundation Models and Their Impact on Healthcare. medRxiv [Preprint]. doi: 10.1101/2025.02.28.25323115

[B22] Leicht E. A. Holme P. Newman M. E. J. (2006). Vertex similarity in networks. Phys. Rev. E 73:026120. doi: 10.1103/PhysRevE.73.026120 16605411

[B23] Masayoshi K. Hashimoto M. Yokoyama R. Toda N. Uwamino Y. Fukuda S. . (2025). EHR-MCP: Real-World Evaluation of Clinical Information Retrieval by Large Language Models via Model Context Protocol.

[B24] Matsumoto N. Moran J. Choi H. Hernandez M. E. Venkatesan M. Wang P. . (2024). KRAGEN: a knowledge graph-enhanced RAG framework for biomedical problem solving using large language models. Bioinformatics 40:btae353. doi: 10.1093/bioinformatics/btae353 38830083 PMC11164829

[B25] Menche J. Sharma A. Kitsak M. Ghiassian S. D. Vidal M. Loscalzo J. . (2015). Uncovering disease-disease relationships through the incomplete interactome. Science 347:1257601. doi: 10.1126/science.1257601 PMC4435741 25700523

[B26] Mesinovic M. Watkinson P. Zhu T. (2025). Explainability in the age of large language models for healthcare. Commun. Eng. 4:128. doi: 10.1038/s44172-025-00453-y 40676176 PMC12271443

[B27] Model Context Protocol (2025). Introduction. Model Context Protocol. Available online at: https://modelcontextprotocol.io/docs/getting-started/intro (Accessed July 20, 2026).

[B28] Morris J. H. Soman K. Akbas R. E. Zhou X. Smith B. Meng E. C. . (2023). The scalable precision medicine open knowledge engine (SPOKE): a massive knowledge graph of biomedical information. Bioinformatics 39:btad080. doi: 10.1093/bioinformatics/btad080 36759942 PMC9940622

[B29] Ng K. K. Y. Matsuba I. Zhang P. C. (2025). RAG in health care: a novel framework for improving communication and decision-making by addressing LLM limitations. NEJM AI 2:AIra2400380. doi: 10.1056/AIra2400380

[B30] OHDSI (2025). OMOP Common Data Model. OHDSI. Available online at: https://ohdsi.github.io/CommonDataModel/ (Accessed October 3, 2025).

[B31] O'Leary A. Fernàndez-Castillo N. Gan G. Yang Y. Yotova A. Y. Kranz T. M. . (2022). Behavioural and functional evidence revealing the role of RBFOX1 variation in multiple psychiatric disorders and traits. Mol. Psychiatry 27, 4464–4473. doi: 10.1038/s41380-022-01722-4 35948661 PMC9734045

[B32] Omar M. Sorin V. Collins J. D. Reich D. Freeman R. Gavin N. . (2025). Multi-model assurance analysis showing large language models are highly vulnerable to adversarial hallucination attacks during clinical decision support. Commun. Med. 5:330. doi: 10.1038/s43856-025-01021-3 40753316 PMC12318031

[B33] Sanda N. Gyori B. M. Quaranta V. Ganguly A. Paul A. (2026). eGoT: enhanced graph-of-thoughts for multi-hop knowledge retrieval and hypothesis generation in biomedicine. Bioinformatics 42(Suppl. 1):btag216. doi: 10.1093/bioinformatics/btag216 42412787 PMC13341121

[B34] Singhal K. Azizi S. Tu T. Mahdavi S. S. Wei J. Chung H. W. . (2023). Large language models encode clinical knowledge. Nature 620, 172–180. doi: 10.1038/s41586-023-06291-2 37438534 PMC10396962

[B35] Soman K. Rose P. W. Morris J. H. Akbas R. E. Smith B. Peetoom B. . (2024). Biomedical knowledge graph-optimized prompt generation for large language models. Bioinformatics 40:btae560. doi: 10.1093/bioinformatics/btae560 39288310 PMC11441322

[B36] Strona G. Nappo D. Boccacci F. Fattorini S. San-Miguel-Ayanz J. (2014). A fast and unbiased procedure to randomize ecological binary matrices with fixed row and column totals. Nat. Commun. 5:4114. doi: 10.1038/ncomms5114 24916345

[B37] Su X. Wang Y. Gao S. Liu X. Giunchiglia V. Clevert D.-A. . (2025). KGARevion: An AI Agent for Knowledge-Intensive Biomedical QA. International Conference on Learning Representations.

[B38] The Book of OHDSI (2021). Observational Health Data Sciences and Informatics. Available online at: https://ohdsi.github.io/TheBookOfOhdsi/ (Accessed July 28, 2026).

[B39] Tu T. Azizi S. Driess D. Schaekermann M. Amin M. Chang P.-C. . (2024). Towards generalist biomedical AI. NEJM AI 1:AIoa2300138. doi: 10.1056/AIoa2300138

[B40] Vatsal S. Dubey H. Singh A. (2026). Agentic AI in healthcare and medicine: a seven-dimensional taxonomy for empirical evaluation of LLM-based agents. IEEE Access 14, 4840–4863. doi: 10.1109/ACCESS.2026.3651218

[B41] Wang Z. Chen Z. Yang Z. Wang X. Jin Q. Peng Y. . (2026). Empowering biomedical evidence exploration and synthesis with deep knowledge graph research. Nat. Mach. Intell. 8, 1142–1156. doi: 10.1038/s42256-026-01266-0 42569156 PMC13449190

[B42] Xiong G. Jin Q. Wang X. Zhang M. Lu Z. Zhang A. (2025). Improving retrieval-augmented generation in medicine with iterative follow-up questions. Biocomputing 2025, 199–214. doi: 10.1142/9789819807024_0015 39670371 PMC11997844

[B43] Xu X. Sankar R. (2025). Large language model agents for biomedicine: a comprehensive review of methods, evaluations, challenges, and future directions. Information 16:894. doi: 10.3390/info16100894

[B44] Yao S. Zhao J. Yu D. Du N. Shafran I. Narasimhan K. . (2023). “ReAct: synergizing reasoning and acting in language models,” in International Conference on Learning Representations.

[B45] Zhao X. Yang M. Tian K. Jiang H. Guo D. Wang Y. . (2026). Performance of latest AI models, RAG, and MCP on lung cancer-related questions. Digit Health 12:20552076261427503. doi: 10.1177/20552076261427503 41836629 PMC12982857

