# Supplementary material for: An agentic AI framework connecting language models to electronic health records and a biomedical knowledge graph for real-world evidence
Source: Front Artif Intell. 2026 Sep 4;9:1883853. doi: 10.3389/frai.2026.1883853 (PMC13585945; doi:10.3389/frai.2026.1883853)
Supplement: Supplementary file 1 [file Data_Sheet_1.pdf]

MedCP Clinical Replication Skill

MedCP Clinical Replication Study Framework

MedCP is an **MCP server** that connects an LLM agent to an **institutional clinical record database (EHR)** and a **large-scale heterogeneous biological knowledge graph**. This skill operationalizes a peer-reviewed paper into a computable protocol, executes EHR + knowledge graph queries through MedCP, performs the paper's statistical analyses (or the closest defensible proxy), and generates a **single Replication Report Artifact** comparing results from the analyzed population with the original publication.

Non-Negotiables

- **No synthetic/simulated data.** Use only data returned by MedCP. If data or access is missing, provide a query/analysis plan instead of fabricating results.
- **No PHI exposure.** Output only **aggregate** statistics appropriate for research reporting. Follow minimum-necessary principles and suppress small cell sizes when required.
- **Keep an audit trail.** Every cohort rule, code set, window, and modeling choice must be recorded so the study can be rerun.
- **Prefer best-effort execution.** If the paper is ambiguous, proceed using standard clinical/epidemiologic conventions and label assumptions explicitly.

Context-Efficient Data Workflow

CRITICAL: Prevent context window exhaustion by never dumping large results into chat.

Principle

For every MedCP extraction: 1. Execute the MedCP query 2. **Save results to a file immediately** 3. Inspect only **metadata** (shape, columns, dtypes) and a small preview 4. Write analysis code that **loads from saved files** 5. Display only **summaries** (counts, effect sizes, tables, plots)

Automatic Thresholds

Result Size	Required Action
≤ 20 rows	OK to display inline
21–1,000 rows	Save to CSV; show schema + `head(5)`
> 1,000 rows	Save to Parquet; show schema + minimal preview
> 100,000 rows	Parquet + chunked extraction (by date range or person_id partitions)

What the Agent Should Display

YES - Row/column counts; schema and dtypes - `head(3–5)` previews only (when permitted) - Summary statistics and model outputs (OR/HR/RR, 95% CI, p-values) - Balance diagnostics (e.g., standardized mean differences) - Plots/visualizations

NO - Full raw query results - Complete DataFrames - Raw intermediate tables with identifying information - Entire file contents

Checkpointing (When Work Becomes Large)

If analysis is complex or long: - Save intermediate "checkpoint" datasets (e.g., `analysis_ready.parquet`) - Save a short status note including: - completed steps - pending steps - key file paths - cohort N and event counts

---

Core Workflow

Execute sequentially. Do not skip Phase 1.

Phase 0: Initialize & Set Scope

1. **Confirm artifacts**
   - Peer-reviewed paper is available as a PDF (or its text).
   - MedCP tool access is available.
2. **Declare replication target**
   - Identify the paper's **primary endpoint** and **primary estimand** (ITT vs as-treated; marginal vs conditional).
   - If multiple cohorts/arms exist, replicate the **main** analysis first.
3. **Select the applicable reporting standard** (see §0A).
4. **Emit the Protocol Header** (see §0B). **This is a gate: do not proceed to Phase 2 until it is complete and has been reviewed by the investigator.**

0A) Reporting Standard Selection (Mandatory)

Identify the design of the paper being replicated and apply the corresponding standards. **RECORD always applies**, because the OMOP instance queried is routinely collected health data.

Design of the source study	Reporting standard	Protocol / design template
Cohort, case-control or cross-sectional (any)	STROBE + **RECORD**	—
Drug or vaccine exposure (any of the above)	STROBE + RECORD + **RECORD-PE**	**HARPER**
Hypothesis-evaluating treatment effect (comparative effectiveness or safety)	**STaRT-RWE** design tables	**HARPER**
Explicit target trial emulation	STROBE + RECORD-PE + STaRT-RWE	**Target trial protocol** (eligibility, treatment strategies, assignment, outcomes, follow-up, causal contrast, analysis plan)

This skill covers observational studies estimating the effect of an exposure on an outcome. Prediction-model development or validation, diagnostic-accuracy studies and Mendelian randomization are **out of scope**: if the source paper is of one of these types, say so and stop rather than forcing it into a cohort design.

If the design fits none of the rows above but remains an effect-estimation study, state which standard is closest and why, and record the deviation. **Do not proceed without selecting one.**

References: - STROBE — von Elm E, et al. *BMJ* 2007;335:806–8. doi:10.1136/bmj.39335.541782.AD - RECORD — Benchimol EI, et al. *PLoS Med* 2015;12:e1001885. doi:10.1371/journal.pmed.1001885 - RECORD-PE — Langan SM, et al. *BMJ* 2018;363:k3532. doi:10.1136/bmj.k3532 - STaRT-RWE — Wang SV, et al. *BMJ* 2021;372:m4856. doi:10.1136/bmj.m4856 - HARPER — Wang SV, et al. *Pharmacoepidemiol Drug Saf* 2023;32:44–55. doi:10.1002/pds.5507 - Target trial emulation — Hernán MA, et al. *JAMA* 2022;328:2446–7. doi:10.1001/jama.2022.21383; Matthews AA, et al. *BMJ* 2022;378:e071108. doi:10.1136/bmj-2022-071108. Known pitfalls: Hubbard RA, et al. *N Engl J Med* 2024;391:1975–7. doi:10.1056/NEJMp2407586

0B) Protocol Header (Mandatory Gate)

Emit **before any query is executed**, and reproduce verbatim as Section 1 of the final report.

Field	Value
Citation (journal, year, DOI)	

Field	Value
Design of source study	
Reporting standard applied (from §0A)	
Setting and calendar timeframe of source study	
Unit of analysis (person / encounter / episode)	
Primary endpoint	
Primary estimand (ITT / as-treated; marginal / conditional)	
**Time zero (index date)**	
Eligibility assessment window	
Baseline lookback window	
Exposure ascertainment window and washout	
Follow-up start (relative to time zero)	
Follow-up end / administrative censoring date	
Censoring: loss to follow-up	
Censoring: death	
Censoring: treatment switch or discontinuation	
Competing risks handling	

Rules: 1. **No field may be left blank.** If a parameter cannot be determined from the source paper, record it as an **explicit assumption** with the convention adopted and the reason. Silence is not permitted. 2. **Immortal-time check.** State where follow-up begins relative to time zero, and confirm that (i) no interval between eligibility and time zero is counted as exposed follow-up, and (ii) no outcome occurring before time zero is counted. 3. **Timeline direction check.** Confirm that every outcome window opens after time zero and that no lookback window extends into the follow-up period. 4. A replication that cannot state its own time zero **fails at Phase 0** and does not proceed to extraction.

Deliverable: completed Protocol Header, reviewed by the investigator before Phase 2.

Phase 1: Deconstruction & Protocol Design

The agent must read the paper and produce a structured protocol blueprint **before querying**.

1A) Cohort Definition

Extract and formalize: - **Inclusion criteria** (age; encounter types; diagnoses; procedures; labs; enrollment/observation time; first-event rules) - **Exclusion criteria** (prior history; contraindications; missingness filters; competing conditions) - **Index date ("time zero")** definition - **Baseline lookback windows** (e.g., −365 to −1 days) - **Exposure ascertainment window** (if applicable) - **Outcome follow-up start**, **censoring**, and **administrative end of study** - **Competing risks** handling (if used)

Deliverable: A cohort specification that can be implemented as query logic.

1B) Variables

For each variable, capture: - Role: **exposure**, **outcome**, **covariate**, **stratifier** - Type: binary / categorical / continuous / time‑varying - Measurement window and aggregation rule (closest pre-index, mean over baseline, etc.) - Paper's exact definition and any thresholds

Deliverable: A variable list with operational definitions.

1C) Methodology

Extract: - Design choices: new-user, active comparator, matching, weighting, lag periods - Statistical model(s): logistic regression, Cox PH, Poisson/neg-bin, linear, mixed models, KM - Adjustment strategy: covariate list; propensity score method; stratification - Missing data handling: complete-case, imputation, "unknown" category - Sensitivity analyses and subgroup analyses - Reported targets: effect sizes, p-values, CIs, and key baseline table rows

Deliverable: A "Model & Estimand" spec matching the paper.

Phase 1B: Variable Mapping Strategy (EHR + Knowledge Graph)

2A) Use the Knowledge Graph for Concept Grounding

Use MedCP's knowledge graph to: - Expand synonyms and clinical phenotype groupings - Map drugs → ingredients/classes (e.g., RxNorm ingredient sets) - Map biomarkers → labs (LOINC and local code mappings) - Link genes/proteins/pathways → clinically measurable proxies (when relevant)

Rule: Code expansion must remain faithful to the paper. Document what was added and why.

2B) Prefer Standard Vocabularies (When Available)

Common mappings (adjust to what MedCP exposes): - Diagnoses: ICD‑10‑CM, SNOMED CT - Procedures: CPT/HCPCS, ICD‑10‑PCS - Labs: LOINC (plus mapped local tests) - Medications: RxNorm ingredient/class; NDC (if available)

If MedCP exposes an **OMOP CDM** layer, use: - `concept`, `concept_ancestor`, `concept_relationship` - Standard concepts where possible (`standard_concept = 'S'`)

2C) Required Mapping Table

Create this mapping plan **before** extraction:

Paper Concept	Operational Definition in Paper	EHR/MedCP Representation	Code System / Tables	Notes

Include windows, thresholds, and hierarchy usage (e.g., "include descendants via concept_ancestor").

2D) Proxy Rules (When Exact Replication Is Impossible)

If a variable cannot be captured exactly: 1. Use the **closest clinically defensible proxy** 2. Explain the difference vs the paper's definition 3. Predict likely bias direction (toward/away from null) 4. Flag it under **Data Availability Limitations**

Phase 2: Data Extraction & Cohort Construction (MedCP)

3A) Incremental Query Strategy

Build in layers:

1. **Cohort discovery**
    - Extract minimal fields: `person_id`, `index_date`, inclusion flags
    - Validate counts (N) and demographic sanity checks
2. **Exposure and outcome ascertainment**
    - Add event dates, follow-up times, censoring indicators
3. **Covariates & baseline characteristics**
    - Add comorbidities, labs/vitals, utilization metrics
4. **Analysis-ready dataset**
    - One row per analysis unit
    - Clean event-time structure for survival models

Performance rule: Pilot on a small time slice first, then scale.

3B) Efficiency Patterns (OMOP-style, if applicable)

Use efficient patterns such as `EXISTS` filters and vocabulary hierarchies.

Drug ingredient capture (example, OMOP):

```sql
SELECT DISTINCT de.person_id, de.drug_exposure_start_date
FROM omop.drug_exposure de
JOIN omop.concept_ancestor ca
  ON de.drug_concept_id = ca.descendant_concept_id
WHERE ca.ancestor_concept_id = :ingredient_concept_id;
```

Condition descendants (example, OMOP):

```
SELECT DISTINCT co.person_id, co.condition_start_date
FROM omop.condition_occurrence co
JOIN omop.concept_ancestor ca
  ON co.condition_concept_id = ca.descendant_concept_id
WHERE ca.ancestor_concept_id = :parent_condition_concept_id;
```

> If MedCP does not accept raw SQL, translate these patterns into the corresponding MedCP query primitives.

3C) Data Quality Checks (Before Modeling)

- Cohort counts: overall; exposed/unexposed; event counts
- Missingness table for key covariates
- Unit harmonization (e.g., mg/dL vs mmol/L)
- Outlier rules (physiologic plausibility when appropriate)
- Duplicates: multiple labs/meds → specify earliest/closest/mean/median rule consistent with paper
- Temporal alignment: verify windows and time zero

Deliverable: A validated analysis dataset saved to file.

Phase 3: Statistical Analysis

4A) Match the Paper's Primary Analysis

Run the same model where feasible: - **Logistic regression** → OR, 95% CI, p-value - **Cox PH** → HR, 95% CI, p-value; check PH assumption if feasible - **Kaplan–Meier** → survival curves, censoring - **Poisson/neg-bin** → rate ratios and offsets - **Matching/weighting** → balance diagnostics + outcome model

If exact replication is not feasible: - Use the closest defensible proxy model - Explain differences and expected impact - Prefer adding a sensitivity analysis if practical

4B) Study Type Decision Guide

- **Time-to-event**: KM + Cox PH (or competing risks if paper uses it)
- **Binary outcome**: logistic regression; consider propensity scores if confounding is central
- **Continuous outcome**: linear or mixed models; repeated measures as needed
- **Comparative effectiveness**: active comparator + new user design when possible; propensity score matching/weighting

4C) Mandatory Diagnostics

Report all of the following. They are not optional and their absence must be explained.

- **Balance:** standardised mean differences for every covariate, before and after matching or weighting. Flag any |SMD| > 0.1.
- **Proportional hazards:** for any Cox model, a test of the PH assumption (Schoenfeld residuals) and the action taken if violated.
- **Event counts:** number of outcome events in each arm, and person-time.
- **Minimum detectable effect:** given the observed number of events, the effect size detectable at 80% power. This is a property of the design, not of the result, and is reported irrespective of the estimate obtained.

4D) Single-Center Power & Interpretation

The analyzed population is drawn from a single health system; expect smaller N and wider CIs than national datasets.

- A wide confidence interval that includes the null is **uninformative**, not confirmatory. A replication whose CI cannot distinguish a null effect from a clinically meaningful one is **compatible with** the original finding, never a **confirmation** of it. State this explicitly.
- Never claim "contradiction" on the basis of an underpowered estimate.
- When the point estimate differs but the CIs overlap, report the overlap and the minimum detectable effect, and draw no inference beyond feasibility.

Phase 4: Validation, Comparison, and Discrepancy Analysis

5A) Side-by-Side Targets

Compare: - Baseline table rows (N, age, sex, key comorbidities, key labs) - Primary effect size(s) and CIs - Absolute risks/incidence (if reported) - Main figure patterns (KM separation; forest plot direction)

5B) Replication Status Rules

Classify each major metric: - **Consistent:** direction matches and CI plausibly overlaps (or significance aligns) - **Partially consistent:** direction matches but magnitude/significance differs materially - **Divergent:** opposite direction or clearly incompatible estimates

5C) Discrepancy Categories (Required)

Every divergence must be assigned to one or more: - **Data Availability:** missing variable, incomplete capture, different coding practice - **Methodological:** index date/window differences, censoring, adjustment set, model mismatch - **Population**

shift: case mix of the analyzed population, referral bias, calendar time, treatment patterns - **Paper ambiguity:** unclear definitions with multiple plausible interpretations

For each, propose at least one remedy: - sensitivity analysis - refined phenotype definition - additional restriction (e.g., outpatient-only, new-user-only) - alternative covariate adjustment approach

---

Required Output: Single Replication Report Artifact

The agent must output **one consolidated report** with these sections (in order):

1. Executive Summary

- Paper: dataset, variables, methodology, key findings
- Replication in the analyzed population: cohort size/timeframe, main model, headline estimate
- Bottom line: replication **successful / partial / divergent**

1b. Protocol Header and Design Table

Reproduce the completed Protocol Header (§0B) verbatim, then the design comparison:

Design element	Original paper	This replication	Deviation and expected effect
Time zero			
Eligibility window			
Exposure ascertainment and washout			
Outcome definition and code set			
Follow-up start and end			
Censoring rules			
Competing risks			
Covariates in adjustment set			
Statistical model			

Every non-empty cell in the **Deviation** column must be carried into the Limitations section.

2. Replication Data Table (Markdown)

Variable / Metric	Original Paper Result	Replication Result	Delta / Status	Notes & Comments

Include baseline characteristics and the primary effect estimate(s). Report, for every effect estimate: **event counts in each arm**, person-time, the point estimate with 95% CI, and the **minimum detectable effect at 80% power** given the observed number of events. An estimate reported without its event count is incomplete.

3. Comparative Visualization (HTML/JS)

- Use **Chart.js** loaded from the **cdnjs.cloudflare.com** CDN
- Replicate the aesthetics of the paper's key figure (KM curve, forest plot, etc.)
- Overlay:
  - **Original Paper** values (hardcoded from paper)
  - **Replication** values (computed)
- Add a caption interpreting overlap/divergence

4. Limitations Analysis

Two separate bullet lists: - **Data Availability Limitations** - **Methodological Limitations**

Also mention generalizability issues (analyzed population vs original setting) when relevant.

Intermediate Result Storage (Mandatory)

Use consistent filenames so reruns are simple:

Phase	Filename Pattern	Format
Concept mapping	`concepts_{domain}.csv`	CSV
Cohort	`cohort_{paper_shortname}.parquet`	Parquet
Baseline	`baseline_characteristics.csv`	CSV
Outcomes	`outcomes_{outcome}.parquet`	Parquet
Analysis-ready	`analysis_ready.parquet`	Parquet
Model outputs	`results_{model}.csv`	CSV

Quality Checks (Before Finalizing)

Protocol gate - [ ] Reporting standard selected (§0A) and named in the report - [ ] Protocol Header complete, no blank fields; undeterminable parameters recorded as explicit assumptions - [ ] Immortal-time check performed and stated - [ ] Timeline direction check performed: no outcome window opens before time zero; no lookback extends into follow-up

Analysis - [ ] Cohort logic matches paper (inclusion/exclusion, windows, time zero) - [ ] Code sets verified (and expansions documented) - [ ] Cohort N and event counts are plausible - [ ] Patient-level proportions verified to lie within [0, 1]; any value above 1 treated as a fatal denominator error - [ ] Missingness assessed and handled per paper (or explained) - [ ] Follow-up time and censoring validated - [ ] Statistical method matches paper (or proxy justified)

Reporting - [ ] Standardised mean differences reported before and after matching - [ ] Proportional-hazards assumption tested for every Cox model - [ ] Event counts and person-time reported for every effect estimate - [ ] Minimum detectable effect at 80% power reported for every endpoint - [ ] Design table completed, with every deviation from the original carried into Limitations - [ ] Replication table filled with targets and notes - [ ] Visualization overlays original vs replication - [ ] Limitations separated into data vs methodology - [ ] No claim of "confirmation" where the confidence interval cannot exclude a clinically meaningful effect

Governance - [ ] No PHI disclosed; small cells handled appropriately - [ ] Large outputs saved to file (no context dumping) - [ ] Protocol Header reviewed by the investigator before extraction

---

Common Pitfalls

1. **Concept mapping too narrow** → use hierarchical expansions cautiously and document them
2. **Index date misalignment** → verify "time zero" and baseline windows rigorously
3. **Censoring mistakes** → define loss to follow-up and administrative censoring clearly
4. **Unit/assay mismatches** → harmonize lab units and reference ranges
5. **Underpowered replication** → interpret non-significance carefully
6. **Surveillance/referral bias** → the analyzed population may differ in case mix from the original cohort
7. **Context exhaustion** → always save files; never paste big tables

---

Usage

A user typically invokes this skill by: 1. Attaching or providing a peer-reviewed paper (PDF or text), 2. Asking to replicate its main analysis using **institutional EHR data via MedCP**, 3. Optionally specifying endpoints, subgroups, or time constraints.

The agent should then: - Deconstruct the paper into a computable protocol - Map variables to EHR/OMOP concepts using the knowledge graph - Extract cohort and variables with MedCP (saving results to files) - Run the matching statistical analysis - Produce the Replication Report Artifact

---

Examples

Prompts that should trigger this skill: - "Here is a paper PDF. Replicate the primary Cox model in our population using MedCP and compare HRs to the paper." - "Use MedCP to reproduce Table 1 baseline characteristics and the main odds ratio in this case-control study." - "Translate the paper's phenotype definitions into ICD-10/LOINC/RxNorm sets using the knowledge graph, then run the replication." - "Replicate the Kaplan–Meier figure by overlaying paper vs replication curves with Chart.js." - "Run the subgroup analysis by sex and age reported in Figure 2 and compare to our population."

Guidelines

- **Governance & privacy:** never output patient-level rows; suppress small cells; avoid verbatim note text unless explicitly allowed and de-identified.
- **Fidelity:** replicate definitions, windows, and estimands; when ambiguous, use standard conventions and label assumptions.
- **Transparency:** document proxies, code sets, and every analytic choice; separate data vs methodological limitations.
- **Rigor:** report uncertainty (95% CI); check model diagnostics where feasible; avoid overclaiming.
- **Scalability:** incremental queries; server-side filtering/aggregation; chunking and file-based workflows.
