# Supplementary material for: An agentic AI framework connecting language models to electronic health records and a biomedical knowledge graph for real-world evidence
Source: Front Artif Intell. 2026 Sep 4;9:1883853. doi: 10.3389/frai.2026.1883853 (PMC13585945; doi:10.3389/frai.2026.1883853)
Supplement: Supplementary file 2 [file Table_2.docx]

**CLINICIAN GRADING RUBRIC**

**Purpose and scope**

This rubric is for grading a “session file” that contains:

a) The natural-language clinical/research question,

b) The agent’s reasoning steps,

c) The executed tool calls (SQL and/or Cypher) used to answer it.

The clinician score reflects correctness and interpretability of the ANSWER with respect to the clinical intent, using the same three-point scale described in the MedCP manuscript:

0 = incorrect or misleading,

1 = mostly correct but with minor deviations from assumptions or modest execution flaws not affecting the overall conclusion,

2 = fully correct, precise, complete, and well-justified.

The clinician should not “grade the writing style”; focus on whether the evidence (queries + results) supports a clinically valid conclusion.

**Required review workflow (clinician)**

1) Identify the question type and the implied study design:

a) Prevalence / “How many have X?” (patient-level count),

b) Incidence / “new diagnosis” (first event in a time window),

c) Treatment uptake / prescribing frequency,

d) Safety signal / adverse event within a window after exposure,

e) Comparative effectiveness / outcomes by regimen,

f) Comorbidity profiling,

g) Mechanistic/biological contextualization using a knowledge graph.

2) Extract the clinical “PICO-like” elements (even if not stated explicitly):

a) Population (age range, sex, disease cohort, setting),

b) Index exposure/intervention (drug/class/procedure) and comparator,

c) Outcomes (clinical codes, labs, visits, hospitalizations),

d) Time horizon (lookback/follow-up),

e) Key exclusions (prior disease, prior exposure, contraindications).

3) Inspect the tool calls to confirm the operational definitions match the clinical intent.

4) Validate that the reported results (counts/rates/ranks/comparisons) are interpretable:

a) Clear numerator and denominator,

b) Absolute counts when feasible (not just percentages),

c) Units and thresholds for labs are clinically sensible.

**Core grading dimensions**

Clinical validity of concept selection (phenotypes, drugs, outcomes)

1) Condition phenotypes:

a) Ensure the condition captured matches the clinical entity (not a symptom, synonym, complication, or overly broad category unless intended).

b) Watch for concept drift where a related-but-different diagnosis is used as the target condition.

2) Drug exposures:

a) Ensure the selected drug(s) truly represent the stated class/strategy (e.g., “triple DMARDs” or “biologic + methotrexate” requires complete and clinically appropriate component definitions).

b) If only a subset of drugs in a class is used, this must be stated; otherwise treat as incomplete.

3) Outcomes/endpoints:

a) Ensure outcomes are clinically appropriate proxies (e.g., a “flare” proxy should be defined and justified; a single nonspecific code may be insufficient).

b) Ensure laboratory thresholds use correct units and do not mix incomparable measurements without harmonization.

Cohort definition and temporal alignment

1) “New users” and “new diagnosis”:

a) Confirm a first-ever exposure/diagnosis definition (or a stated lookback period) is used.

2) Time windows:

a) Confirm outcomes are measured AFTER the index date within the stated follow-up window (e.g., 30/90/180 days).

b) Confirm “past year / last 24 months / past two years” is implemented accurately (no future windows, no unbounded history unless intended).

3) Activity/observability:

a) If the question implies “currently active in our records,” ensure some observation period constraint or recent encounter evidence is used (or the limitation is clearly acknowledged).

Interpretability and reporting quality

1) Minimum reporting elements for a high score:

a) Cohort size (denominator),

b) Outcome counts (numerator) and a proportion/rate,

c) Clear timeframe,

d) If comparing groups: show results for both groups and the comparison metric (difference/ratio), even if descriptive.

2) Penalize (usually to score 1) if:

a) Only relative statements are given without any counts,

b) The answer is not structured in the way the question asks (e.g., asked “by indication” but grouped differently),

c) Interpretation is generic and could be misread as a clinical guideline rather than descriptive EHR evidence.

Biological contextualization using the knowledge graph

1) If the question requests biological mechanisms/pathways:

a) The mechanistic claims must be supported by KG tool calls OR clearly labeled as general background not derived from the KG.

2) Hallucination rule (important):

a) If the answer attributes a specific fact to the KG that was not retrieved by KG queries, treat as misleading and score down (often to 1; to 0 if it materially changes the conclusion).

3) Clinical–biological coherence:

a) Mechanisms should plausibly connect the clinical entities (disease ↔ pathway ↔ drug target) and not contradict established clinical knowledge.

Data adequacy, limitations, and plausibility checks

1) Clinicians should sanity-check magnitudes and plausibility:

a) Extremely low event counts for common outcomes may reflect under-capture or wrong phenotype; acceptable only if explicitly acknowledged and interpretation is cautious.

b) Implausible time ranges (e.g., events counted into future years) are major errors.

2) Missingness/coverage:

a) If key data elements are likely missing in OMOP (outside labs, non-standard panels, incomplete medication capture), a strong answer notes this limitation.

3) “Reasoning correct but no data”:

a) If the workflow never actually retrieves the needed clinical outcomes (e.g., only discusses mechanism), the question is not answered.

**Scoring rules (0/1/2) for the Clinician**

Score = 2 (fully correct, precise, well-justified)

Assign 2 only if ALL are true:

1) The operational cohort/exposure/outcome definitions match the clinical intent and timeframe.

2) The answer is evidence-backed by the executed queries (EHR and KG as applicable).

3) Results are interpretable:

a) Includes cohort size and outcome counts/rates for the requested window,

b) Provides both arms for comparative questions (or clearly states when data are insufficient).

4) Any important EHR/KG limitations or data sparsity that could bias interpretation are disclosed.

5) Biological contextualization (if requested) is coherent and either query-supported or clearly labeled as background.

Score = 1 (mostly correct but suboptimal)

Assign 1 if the answer is broadly correct, but has ONE OR MORE minor issues that do not overturn the main conclusion:

1) Incomplete capture:

a) Incomplete medication list for a stated class,

b) Incomplete set of outcomes/complications when asked for “most frequent” categories.

2) Minor clinical mismatch:

a) Includes a closely related but not exact phenotype (e.g., a synonym counted as a “comorbidity”),

b) Uses a reasonable but imperfect proxy outcome that could misclassify at scale.

3) Reporting/interpretation shortcomings:

a) Missing absolute counts (only percentages or qualitative statements),

b) Generic contextualization that risks misinterpretation (but results are still correct).

4) KG support is partial:

a) Mechanism discussion is plausible but not tightly grounded in the KG queries,

b) A minor KG attribution error that does not change the overall conclusion.

Score = 0 (incorrect or misleading)

Assign 0 if ANY major issue is present:

1) Clinically wrong concept selection (e.g., drug/condition relationship is incorrect or contradicts basic clinical knowledge) and it affects the conclusion.

2) Major cohort/time-window error:

a) Wrong timeframe (e.g., includes future years or fails to apply “past year”),

b) Key restriction omitted (e.g., required subtype such as HBeAg-positive, organism-specific infections, or etiological agent).

3) Failure to answer:

a) No outcome rates/comparisons are actually computed for the asked window,

b) Output is hypothetical/speculative rather than query-driven.

4) Misleading mechanistic claims:

a) Attributes key evidence to the KG without actually retrieving it, and this drives the conclusion.
