# Supplementary material for: An agentic AI framework connecting language models to electronic health records and a biomedical knowledge graph for real-world evidence
Source: Front Artif Intell. 2026 Sep 4;9:1883853. doi: 10.3389/frai.2026.1883853 (PMC13585945; doi:10.3389/frai.2026.1883853)
Supplement: Supplementary file 3 [file Table_3.docx]

**BIOINFORMATICIAN GRADING RUBRIC**

**Purpose and scope**

This rubric is for grading a single markdown “session file” that contains:

a) The natural-language question,

b) The agent’s reasoning steps, and

c) One or more executed tool calls (SQL and/or Cypher).

The goal is to score the DATABASE QUERIES (not the prose) for whether they correctly and efficiently answer the intended information need. Output is a single score per file: 0 (incorrect/misleading), 1 (mostly correct but suboptimal), or 2 (immaculate).

**Required review workflow**

1) Read the question and write down (mentally or on scratch) the exact intent:

a) Population/cohort definition (who is included/excluded),

b) Exposure(s) / condition(s) / measurement(s) of interest,

c) Outcome(s) / endpoints,

d) Time windows (index date, lookback, follow-up),

e) Unit/threshold requirements (labs, vitals),

f) Stratifiers (age, sex, site, era) and the expected level of aggregation (patient vs visit vs event).

2) Extract EVERY executed tool call (SQL and/or Cypher) and review each query end-to-end.

3) Classify each query as either:

a) Exploratory / vocabulary-discovery (allowed to be broad; used to find concepts or sanity-check),

b) Intermediate cohort-building (creates lists/CTEs to define cohort/exposures),

c) Final analytic query (the query that produces the reported counts/rates/ranks/comparisons).

4) Score the file based primarily on the FINAL analytic query(ies), while considering whether exploratory steps were refined appropriately.

**Core grading dimensions**

Intent alignment (scope and cohort logic)

1) The final query must implement the question’s key inclusion/exclusion criteria.

2) Check for scope drift:

a) The final query counts exposures or diagnoses without joining to the intended cohort table/CTE.

b) The query answers a different time window than asked.

c) The query switches from “patients” to “events/rows” without justification.

3) Required examples of correct alignment:

a) “New users” questions require a first-exposure definition (e.g., MIN(drug_exposure_start_date) per person and restricting outcomes to a window after that date).

b) “Within X days/months” requires a date predicate anchored to an index date, not just filtering calendar dates.

Vocabulary / ontology use (OMOP and clinical terminologies)

1) Exploratory string matching (e.g., LIKE '%asthma%') is acceptable for early concept discovery.

2) Final counting/aggregation SHOULD be vocabulary-driven:

a) Prefer concept_ancestor to include descendants of a condition/drug/lab concept.

b) Avoid final reliance on concept_name LIKE filters when a hierarchical mapping is expected.

3) When a fixed list of concept_ids is used in the final query, verify it is justified:

a) It must be complete for the stated phenotype/class OR explicitly stated as a non-exhaustive subset.

b) If the question asks “any statin” or “type 2 diabetes”, ancestor expansion is usually expected.

Temporal logic (dates, index dates, windows, and person-time)

1) Verify date functions match the SQL dialect (SQL Server/T-SQL):

a) Use DATEADD/DATEDIFF/GETDATE/SYSDATETIME; avoid Postgres-only functions (CURRENT_DATE, AGE()) unless clearly supported.

2) Check that:

a) The correct anchor date is used (e.g., index exposure date, diagnosis date, visit date),

b) Lookback and follow-up windows match the question (e.g., 30/90/180 days),

c) “Past year / past two years / last 24 months” is implemented correctly (not future dates or unbounded ranges),

d) Observation period constraints are applied when needed (to ensure “active in records” or “currently observable”).

Counting, aggregation, and de-duplication

1) Confirm what is being counted:

a) COUNT(DISTINCT person_id) for patient-level prevalence,

b) COUNT(DISTINCT visit_occurrence_id) for visit-level counts,

c) COUNT(*) for raw events only when events are the intended unit.

2) If reporting percentages or prevalences:

a) Denominator must match the cohort size,

b) Values >100% strongly suggest row-multiplication or wrong denominator and are usually a fatal error.

3) Check joins that create multiplicative rows:

a) Joining condition_occurrence to drug_exposure and measurement without controlling multiplicity,

b) Missing DISTINCT or missing windowing (ROW_NUMBER / MIN date) where required.

4) For “top comorbidities”:

a) Exclude the index condition itself and obvious duplicates/synonyms where possible,

b) Ensure comorbidity is defined at patient level, not condition rows.

SQL/Cypher correctness (syntax and schema correctness)

1) SQL:

a) Must be executable (no missing aliases, dangling columns, placeholder text, or invalid nested subqueries),

b) Must reference appropriate OMOP tables/columns for the task (condition_occurrence, drug_exposure, measurement, visit_occurrence, person, observation_period, etc.).

2) Cypher:

a) Must be executable read-only Cypher (no CREATE/MERGE/SET/DELETE),

b) Must avoid cartesian products by linking patterns and using WITH appropriately,

c) Aggregations must be logically correct (COUNT(DISTINCT …) where needed),

d) Ensure MATCH patterns align with intended node labels/relationships and the queried property keys exist.

Efficiency and robustness (suboptimal vs unacceptable)

1) A query can be correct but suboptimal:

a) Excessive concept_name LIKE scans in the final step when concept_ancestor would be straightforward,

b) Selecting many unused columns, repeated subqueries, or unnecessary DISTINCTs that don’t change results,

c) Large cartesian joins or repeated scans without filters.

2) Efficiency is “Poor” when the query is likely to time out or scale badly in an OMOP-sized database:

a) Leading-wildcard LIKE ('%term%') on concept_name in final cohort selection without narrowing,

b) Cartesian products (missing join predicates),

c) Repeated full-table scans when an indexed concept_id path is available.

**Scoring rules (0/1/2) for the Bioinformatician**

Score = 2 (immaculate)

Assign 2 only if ALL are true:

1) The final analytic query(ies) answer the question exactly (cohort, exposure/outcome, stratifiers, time window).

2) Syntax is valid for the dialect (SQL Server/T-SQL and Neo4j Cypher as applicable).

3) Ontology/vocabulary use is appropriate:

a) Exploratory LIKE is fine early, but the final counting step is hierarchy-driven when expected.

4) De-duplication is correct (no inflation from joins; denominators and DISTINCT usage are correct).

5) Query is reasonably concise and efficient; any redundancy is harmless and cannot plausibly change results.

Score = 1 (mostly correct but suboptimal)

Assign 1 if the queries basically answer the intent, but ONE OR MORE minor issues exist that could affect results at scale or reduce robustness:

1) Minor cohort/phenotype mismatch:

a) Uses concept_name LIKE in the final step instead of concept_ancestor (risking missed descendants/synonyms),

b) Uses a plausible but incomplete concept_id list for a broad class without justification.

2) Minor counting risk:

a) Counts events instead of patients but the narrative still reflects the intended question,

b) Slightly brittle joins that could duplicate rows, but likely limited by DISTINCT elsewhere.

3) Minor temporal assumption:

a) Uses an approximate age calculation or slightly different date anchor that likely doesn’t flip the conclusion.

4) Inefficiency without changing meaning:

a) Obvious more efficient equivalent exists (e.g., replace long CASE+LIKE drug classification with ancestor mapping),

b) Uses unnecessary intermediate queries in the final step.

Score = 0 (incorrect or misleading)

Assign 0 if ANY major issue is present:

1) Invalid/unexecutable SQL or Cypher (undefined alias, placeholder text, broken syntax).

2) Wrong schema/tables/joins such that the cohort or outcome is not what the question asks.

3) Major scope drift:

a) Final query does not restrict to the cohort of interest,

b) Key inclusion/exclusion criteria are missing (e.g., “new users” not enforced; required subtype restriction ignored).

4) Major counting error:

a) Row multiplication leading to impossible prevalences (>100%) or otherwise clearly wrong magnitudes,

b) No DISTINCT where required; numerator/denominator mismatch.

5) The file provides no executable query for the key analytic step, or results are hypothetical/not query-driven.

**Tie-breaking and edge cases**

1) If a question is clinically ambiguous (e.g., “active disease” not defined), use the reasoning steps in the file to infer intended operationalization; score down only if the implemented definition contradicts both the question and the reasoning.

2) If the dataset appears sparse and the query returns few/zero events:

a) Do not penalize purely for low counts if the query is correct.

b) Do penalize if the agent claims results without actually querying (or if the query cannot produce the claimed result).

3) If both SQL and Cypher are used:

a) Score based on whether the combined workflow answers the question (e.g., KG identifies a set; EHR quantifies it).

b) If either side is broken in a way that changes the answer, score accordingly.
