# Supplementary material for: An agentic AI framework connecting language models to electronic health records and a biomedical knowledge graph for real-world evidence
Source: Front Artif Intell. 2026 Sep 4;9:1883853. doi: 10.3389/frai.2026.1883853 (PMC13585945; doi:10.3389/frai.2026.1883853)
Supplement: Supplementary file 4 [file Table_4.docx]

# **Supplementary Table 1. *Criteria used to assign benchmark questions to complexity tiers.***

The 100 benchmark questions were assigned to one of ten complexity tiers before any MedCP execution, based on the following four criteria. For each question, the tier was selected as the highest tier whose criteria were jointly satisfied. Tier labels and exemplar questions are also reported in the main-text **Table 2**.

| **Tier** | **Scope** | **Min. expected tool calls** | **Reasoning depth** | **Cross-source integration required** | **Statistical / analytical method expected** |
| --- | --- | --- | --- | --- | --- |
| 1 | Baseline size of a population or exposure | 1 | Direct lookup | EHR only | Counts |
| 2 | Frequencies with one simple stratifier or a small predefined set | 1–2 | Direct lookup with stratification | EHR only | Counts and proportions |
| 3 | Event rates after a new exposure using routine outcomes | 2–3 | New-user / cohort definition | EHR only | Incidence proportion, simple rates |
| 4 | Temporal trends and epoch comparisons | 2–3 | Time-series aggregation | EHR only | Trend description, optional seasonal decomposition |
| 5 | Concept ranking and biological contextualization | 2–4 | Ranking with light biological context | EHR (primary) ± KG (optional) | Counts, proportions, optional KG annotation |
| 6 | Comorbidity profiling, condition co-occurrence, and biological linkage | 3–6 | Multi-cohort analysis with KG-based linkage | EHR + KG | Co-occurrence, KG path or shared-feature analysis |
| 7 | Disease–disease relationships grounded in molecular similarity | 4–8 | Cross-source synthesis of clinical and molecular evidence | EHR + KG | KG similarity / overlap + EHR validation |
| 8 | Drug-class effects requiring mechanistic stratification | 4–8 | Mechanism-aware cohort comparison | EHR + KG | Stratified rates, drug–target mapping |
| 9 | Within-cohort multi-outcome comparison across regimens / classes | 5–10 | Comparative effectiveness over a defined window | EHR (primary) ± KG (mechanistic) | Time-to-event or repeated-measures comparison |
| 10 | Most demanding descriptive comparisons (incident or transition designs) | 6–12 | Multi-step inferential with explicit windows and control sets | EHR + KG | Survival or time-series, mechanism-aware contrasts |

Notes:

• The "Min. expected tool calls" column reflects the minimum number of distinct EHR or KG queries we anticipated as necessary to construct an answer, not the actual number of tool calls observed at run time. Observed tool-call counts are reported in Supplementary Data 1.

• Tiers 1–5 are dominated by EHR-only operations and were expected to be answerable without knowledge-graph traversal; tiers 6–10 require either explicit cross-source integration or mechanistic reasoning grounded in the biomedical knowledge graph. This a priori expectation is the basis of the tier-stratified comparisons reported in the main text.
