# Supplementary material for: An agentic AI framework connecting language models to electronic health records and a biomedical knowledge graph for real-world evidence
Source: Front Artif Intell. 2026 Sep 4;9:1883853. doi: 10.3389/frai.2026.1883853 (PMC13585945; doi:10.3389/frai.2026.1883853)
Supplement: Supplementary file 5 [file Table_5.docx]

# **Supplementary Table 2. *Model-agnostic failure modes across EHR, knowledge-graph, and combined MedCP workflows.***

Recurring operational and analytical failure patterns were summarized from the physician and bioinformatician notes for the current 100-question, two-model benchmark. The taxonomy is organized by the tool or workflow layer at which the weakness arises; model-specific differences are treated as differences in frequency, not as distinct failure mechanisms.

| **Tool / workflow layer** | **Model-agnostic weakness** | **Operational signature** | **Representative cross-model evidence** | **Consequence and safeguard** |
| --- | --- | --- | --- | --- |
| **EHR / SQL** | Incomplete temporal cohort design | First-recorded exposure treated as incident use without an observable washout; index dates and outcomes were misaligned; complete follow-up or baseline outcome exclusion was not required. | Q3.8 (new TMP-SMX use and potassium), Q9.3 (new AF and initial anticoagulation), and Q10.1 (dopamine-agonist side effects) were submaximal in all 4/4 model-configuration runs. | Can bias incidence and temporal attribution. Require observation-period coverage, prespecified washout, index alignment, complete follow-up, and exclusion of prevalent outcomes. |
| **EHR / SQL** | Overlapping aggregation or ill-defined denominators | Raw parent/child diagnosis codes were ranked as separate phenotypes, the same person could contribute to overlapping categories, or raw event counts were compared without exposed or tested denominators. | Q5.1 ranked diagnosis records rather than distinct women, Q5.2 fragmented parent/child cancer sites, and Q6.3 used overlapping comorbidity groups; all three questions were submaximal in all 4/4 model-configuration runs. | Can fragment or inflate rankings and confound signals with use/testing intensity. Roll codes into mutually exclusive phenotypes, count distinct patients once per category, and report group-specific denominators. |
| **EHR / SQL** | Exposure, outcome, unit, or indication misclassification | Combination products were defined by either component; same-day ingredient records were treated as sustained regimens; laboratory thresholds lacked unit validation; or drug use and outcomes were not tied to the clinical index. | Q3.8 could admit trimethoprim or sulfamethoxazole monotherapy; Q9.3 could classify prophylactic/bridging heparin as AF therapy; Q10.1 counted common pre-existing Parkinson manifestations. All were submaximal in 4/4 runs. | Can create non-comparable groups or false outcomes. Use ingredient and exposure-overlap logic, validated units/phenotypes, incident-regimen washout, and indication/index-date validation. |
| **Knowledge graph** | Incomplete graph traversal or ontology coverage | String matching, fixed identifiers, or hand-selected examples substituted for complete, relation-specific graph traversal; broad shared-gene/pathway links were sometimes treated as specific mechanistic support. | For Q7.6 (pathway-defined rheumatoid-arthritis drug classes) and Q7.8 (electrolyte-associated drug classes), both KG-enabled models were submaximal (2/2 each) after eligible graph-derived sets were manually substituted, incompletely retrieved, or used at inconsistent granularity. | Can omit eligible entities or overstate nonspecific biology. Enforce relation- and provenance-aware traversal, hierarchy completeness checks, and explicit separation of graph evidence from model prior knowledge. |
| **Knowledge graph** | Graph-query or retrieval incompleteness | Cypher/API timeouts, truncated result sets, or unresolved graph schema/edge choices prevented the requested entity or mechanism set from being completed before interpretation. | This occurred in both model families, including incomplete class/mechanism retrieval in Q7.8 and other high-tier cross-source questions; frequency and severity differed by model and run. | Can turn a graph-grounded task into a partial or parametric answer. Add bounded retry/resume behavior, schema-aware query templates, result-completeness checks, and explicit disclosure when KG evidence is unavailable. |
| **Combined KG → EHR** | Incomplete entity mapping and set transfer | KG-returned diseases, drugs, mechanisms, or classes were mapped to incomplete OMOP concept lists; some graph entities were dropped, manually reconstructed, or mapped at inconsistent levels of granularity. | Q7.6 and Q7.8 exposed this cross-source seam in both KG-enabled models (2/2 each): graph-defined classes were not consistently transferred into complete, comparable EHR cohorts. | Can make a biologically valid graph result yield a biased clinical ranking. Persist the full KG result set, use auditable crosswalks and coverage reports, and stop when mapping completeness falls below a prespecified threshold. |
| **Combined KG + EHR** | Incomplete multi-part synthesis | The workflow completed only the EHR count or only the mechanistic narrative, or interpreted literature/model knowledge as if it were supported by both executed data sources. | Q6.3 (comorbidity plus shared pathways), Q7.8 (KG classes plus 30-day EHR outcomes), and Q10.6 (clinical comparison plus immunologic explanation) were submaximal in both KG-enabled models; the paired EHR-only runs failed the parallel completeness requirement on the same questions. | Can produce a plausible answer without the requested cross-source evidence. Track each requested component, require source-specific evidence before synthesis, and run a final completeness/provenance checklist. |
| **Cross-cutting analysis** | Unsupported comparative or causal interpretation | Sparse, imbalanced, or unadjusted descriptive rates were presented as class differences without uncertainty, formal comparison, or adequate confounding control. | Q8.9 (checkpoint-inhibitor classes) and Q10.6 (COPD maintenance approaches) were submaximal in all 4/4 runs despite generally plausible clinical interpretations. | Can overstate unstable or non-comparable estimates. Label results as descriptive, report uncertainty and cell sizes, and use an adjusted design before causal language. |
| **Cross-cutting orchestration** | Timeout, truncation, or scope loss | A tool/API failure or long multi-step trace ended after a partial count, omitted a requested component, or substituted an expected narrative for an executed result. | Examples occurred in both model families and in EHR-only and KG-enabled workflows; they were more frequent in Claude Opus 4.8 runs, showing that frequency can be model-dependent even when the weakness is not. | Can yield no answer or an unaudited partial answer. Use resumable checkpoints, bounded retries, explicit completion criteria, and a final checklist covering every requested result component. |

Notes:

• A "submaximal" run was defined as a mean physician/bioinformatician score below 2. Of 400 model-configuration runs, 295 were submaximal: 60 GPT-5.5 with KG, 80 GPT-5.5 without KG, 71 Claude Opus 4.8 with KG, and 84 Claude Opus 4.8 without KG.

• At the question level, 91/100 were submaximal in at least one run, 54/100 in both models with KG, 74/100 in both models without KG, and 49/100 in all four model-configuration runs; "4/4" denotes that last cross-model occurrence. Failure families are qualitative and non-exclusive, so rows should not be summed. A model may exhibit a given weakness more often, but each row describes a system-level failure that can occur with either model; full per-run scores and reviewer annotations are provided in Supplementary Data 1. Examples illustrate mechanisms rather than category prevalence. The with-KG bioinformatician scores were regraded; without-KG scores retained their original grading.
