# Supplementary material for: An agentic AI framework connecting language models to electronic health records and a biomedical knowledge graph for real-world evidence
Source: Front Artif Intell. 2026 Sep 4;9:1883853. doi: 10.3389/frai.2026.1883853 (PMC13585945; doi:10.3389/frai.2026.1883853)
Supplement: Supplementary file 6 [file Table_6.docx]

**Supplementary Table 3. MedCP in the landscape of frameworks and tools for clinical databases and biomedical KG access.**

Representative recent systems and the established OMOP/OHDSI tooling, described by their approach, clinical-data access, biomedical-knowledge integration, interface and query auditability, demonstrated evaluation, and availability.

| **System** | **Scope / intended use** | **Approach (agentic character; model access)** | **Clinical / EHR data access** | **Biomedical knowledge integration** | **Interface and query auditability** | **Demonstrated evaluation** | **Availability / reference** |
| --- | --- | --- | --- | --- | --- | --- | --- |
| **MedCP** | Research assistant for real-world-evidence generation and hypothesis generation: cross-source EHR + knowledge-graph exploration and replication of published observational studies | LLM-agentic via MCP (open standard); the 100-question benchmark used GPT-5.5 and Claude Opus 4.8 through the secure UCSF endpoint, and the case-study analyses used Claude Opus 4.5, so results are model- and deployment-specific. | Institution-wide UCSF OMOP CDM (>7M patients); synthetic OMOP (Eunomia) for reproducible testing | SPOKE (~45M nodes, ~133M edges instance); queried jointly with the EHR in one session | Natural-language chat; every step emits explicit SQL/Cypher preserved in the run log for review | 400 model-configuration runs across 100 questions and 10 complexity tiers; two-model BiomixQA evaluation; two published-study feasibility replications. | GitHub/source code |
| EHR-MCP | EHR information retrieval / clinical Q&A over hospital records | LLM-agentic; custom MCP server (GPT-4.1 ReAct agent) | Real, single-site hospital EHR (Keio University Hospital); 8 patients (infection-control cases); direct database access, not standardized to FHIR/OMOP | — | Natural-language; MCP tool calls | 6 information-retrieval tasks completed correctly | Preprint. Masayoshi K, Hashimoto M, Yokoyama R, et al. (2025). EHR-MCP: real-world evaluation of clinical information retrieval by LLMs via Model Context Protocol. arXiv:2509.15957. doi:10.48550/arXiv.2509.15957 |
| MCP-FHIR | Clinical decision support over FHIR resources | LLM-agentic; MCP over the FHIR API | Synthetic FHIR R4 resources (SMART-on-FHIR sandbox); patient/element counts not reported | — | Natural-language; standards-based FHIR calls | Clinical decision-support tasks (framework demonstration) | Open-source. Ehtesham A, Singh A, Kumar S (2025). Enhancing clinical decision support and EHR insights through LLMs and the Model Context Protocol: an open-source MCP-FHIR framework. arXiv:2506.13800. doi:10.48550/arXiv.2506.13800 |
| MedAgentBench | Benchmark for evaluating clinical LLM agents (evaluation harness, not a deployed tool) | LLM-agentic; tool-use benchmark | Real de-identified EHR (Stanford STARR): 100 patient profiles, >700K FHIR elements (563K Observation, 125K Procedure, 75K Condition, 22K MedicationRequest), served via a virtual FHIR environment | — | Natural-language agent tasks | 300 physician-written tasks across 10 categories | Open (GitHub). Jiang Y, Black KC, Geng G, et al. (2025). MedAgentBench: a realistic virtual EHR environment to benchmark medical LLM agents. arXiv:2501.14654. doi:10.48550/arXiv.2501.14654 |
| KGT | Biomedical question answering / KG-grounded reasoning | KG-augmented LLM reasoning / prompting | — | SmartQuerier Oncology KG (SOKG): 3.64M nodes (24 entity types), 10.66M edges (21 relation types) — genes/mutations, drugs, cancer types, biomarkers, trials; source databases not named in the paper | Natural-language question answering | PcQA benchmark (405 QA pairs) | Feng Y, Zhou L, Ma C, et al. (2025). Knowledge graph-based thought: a knowledge graph-enhanced LLM framework for pan-cancer question answering. GigaScience 14:giae082. doi:10.1093/gigascience/giae082; PMID 39775838 |
| KGARevion | Knowledge-intensive biomedical question answering | LLM-agentic; KG-augmented agent | — | PrimeKG (~129K nodes/10 types; ~4.05M edges/30 types; 20 sources incl. DisGeNET, DrugBank, GO, HPO, MONDO, Reactome; 17,080 diseases) + OGB-biokg | Natural-language question answering | MedQA, MMLU-Med, PubMedQA, BioASQ, MedDDx, AfriMed-QA | Su X, Wang Y, Gao S, et al. (2025). KGARevion: an AI agent for knowledge-intensive biomedical QA. ICLR 2025. arXiv:2410.04660 |
| i-MedRAG | Medical exam / clinical question answering (USMLE, MedQA) | Iterative retrieval-augmented generation (follow-up querying) | — | — (text-RAG over MedRAG corpora: Textbooks + StatPearls, MedCPT retriever; not a knowledge graph) | Natural-language question answering | MedQA (USMLE), MMLU-Med | Xiong G, Jin Q, Wang X, et al. (2025). Improving retrieval-augmented generation in medicine with iterative follow-up questions. Pac Symp Biocomput 30:199–214. doi:10.1142/9789819807024_0015; PMID 39670371 |
| KG-RAG | Biomedical question answering; improving LLM factuality | RAG with knowledge-graph-based prompt generation | — | SPOKE (42M nodes/28 types, 160M edges/91 types, 41 databases);  >87K nodes embedded for the disease proof-of-concept | Natural-language question answering | Improved LLM factuality on graph-grounded medical questions | Soman K, Rose PW, Morris JH, et al. (2024). Biomedical knowledge graph-optimized prompt generation for large language models. Bioinformatics 40(9):btae560. doi:10.1093/bioinformatics/btae560; PMID 39288310 |
| KRAGEN | Biomedical problem-solving question answering | KG-enhanced RAG using graph-of-thoughts decomposition | — | AlzKB (Alzheimer's Knowledge Base, alzkb.ai) — genes, diseases, drugs and related entities; scale not reported | Natural-language question answering | Biomedical problem-solving QA | Matsumoto N, Moran J, Choi H, et al. (2024). KRAGEN: a knowledge graph-enhanced RAG framework for biomedical problem solving using large language models. Bioinformatics 40(6):btae353. doi:10.1093/bioinformatics/btae353; PMID 38830083 |
| ATLAS / CIRCE (OHDSI) | Cohort definition for observational research | Deterministic, non-LLM; graphical cohort definition with generated SQL | Any OMOP CDM; portable across the OHDSI federated network | — | Graphical/programmatic; fully specified, versionable, shareable cohort definitions | Community-standard tooling underpinning large-scale, validated observational and network studies | Open source (OHDSI). Hripcsak G, Duke JD, Shah NH, et al. (2015). Observational Health Data Sciences and Informatics (OHDSI). Stud Health Technol Inform 216:574–578. doi:10.3233/978-1-61499-564-7-574; PMID 26262116. The Book of OHDSI (2021). doi:10.5281/zenodo.4265256 |
| HADES (OHDSI) | Population-level analytics (estimation, prediction) for observational research | Deterministic, non-LLM; standardized R analytics packages | Any OMOP CDM | — | R / programmatic; standardized, reproducible analytic pipelines | Validated population-level estimation and patient-level prediction across the OHDSI network | Open source (OHDSI). Hripcsak G, Duke JD, Shah NH, et al. (2015). Observational Health Data Sciences and Informatics (OHDSI). Stud Health Technol Inform 216:574–578. doi:10.3233/978-1-61499-564-7-574; PMID 26262116. The Book of OHDSI (2021). doi:10.5281/zenodo.4265256 |
